# Individual heterogeneity in life histories and eco-evolutionary dynamics

**DOI:** 10.1111/ele.12421

**Published:** 2015-03-23

**Authors:** Yngvild Vindenes, Øystein Langangen

**Affiliations:** 1 Centre for Ecological and Evolutionary Synthesis (CEES), Department of Biosciences, University of OsloOslo, Norway

**Keywords:** Demographic heterogeneity, eco-evolutionary response, evolutionary demography, individual differences, structured population

## Abstract

Individual heterogeneity in life history shapes eco-evolutionary processes, and unobserved heterogeneity can affect demographic outputs characterising life history and population dynamical properties. Demographic frameworks like matrix models or integral projection models represent powerful approaches to disentangle mechanisms linking individual life histories and population-level processes. Recent developments have provided important steps towards their application to study eco-evolutionary dynamics, but so far individual heterogeneity has largely been ignored. Here, we present a general demographic framework that incorporates individual heterogeneity in a flexible way, by separating static and dynamic traits (discrete or continuous). First, we apply the framework to derive the consequences of ignoring heterogeneity for a range of widely used demographic outputs. A general conclusion is that besides the long-term growth rate lambda, all parameters can be affected. Second, we discuss how the framework can help advance current demographic models of eco-evolutionary dynamics, by incorporating individual heterogeneity. For both applications numerical examples are provided, including an empirical example for pike. For instance, we demonstrate that predicted demographic responses to climate warming can be reversed by increased heritability. We discuss how applications of this demographic framework incorporating individual heterogeneity can help answer key biological questions that require a detailed understanding of eco-evolutionary dynamics.

## Introduction

Intraspecific variation in properties affecting fitness is ubiquitous in natural populations, including morphological traits, behavioural traits and impacts of environmental factors like spatial location (Kendall & Fox 2002; Vindenes *et al*. 2008; Bolnick *et al*. 2011). Such variation is an essential part of evolutionary theory, whereas in population ecology it is often ignored as the focus is typically on population-level (average) processes. Recently, however, the role of individual variation in shaping such ecological processes has received increasing interest (Wilson & Nussey 2010; Bolnick *et al*. 2011; de Valpine *et al*. 2014), and studies on eco-evolutionary dynamics have emphasised the link between ecological and evolutionary processes on a contemporary time scale (Schoener, 2011; Smallegange & Coulson 2013). An increased understanding of the underlying mechanisms of eco-evolutionary dynamics is essential to answer many of the currently important questions in biology, such as how fast species will adapt to environmental impacts (Pelletier *et al*. 2007; Smallegange & Coulson 2013).

Demographic population models like matrix models (Leslie, 1945; Lefkovitch, 1965; Caswell, 2001) and integral projection models (IPM; Easterling *et al*. 2000; Ellner & Rees 2006) connect individual-level processes like survival and reproduction, and population-level parameters describing the dynamics and average life history (Caswell & John 1992). However, the majority of applications of these models assume that individuals follow the same life-history trajectory, ignoring underlying individual differences. Classical theory for age structured populations provide useful descriptions of the average life history of a species, and have been successfully applied to explain interspecific differences (Fisher, 1930; Charlesworth, 1994; Roff, 1996). Nonetheless, the average life history does not necessarily provide a good description of individual life histories.

A well-known example that a population-level average may not represent individual properties is the so-called ‘frailty effect’, referring to an apparent effect of reduced mortality over age that is actually caused by individual heterogeneity in survival (Vaupel *et al*. 1979). Thus, at the individual level, mortality may not be changing with age but the average mortality is. This effect of individual heterogeneity has important implications for our understanding of senescence (Vaupel & Yashin 1985; Loison *et al*. 1999; Caswell, 2014). Similar mechanisms can shape any parameter of interest calculated for the average life history. For instance, in a study of monocarpic plants Rees *et al*. (2000) showed that the optimum flowering size as predicted from the average life history is not a good predictor of the optimum size in individual plants.

Another important consequence of heterogeneity is that demographic outputs characterising population dynamics and life history (e.g. population growth rates, net reproductive rate, generation time and extinction risk) can be biased if the heterogeneity is not recognised and accounted for. Studies exploring such effects of unobserved heterogeneity have so far mainly focused on the effects on demographic stochasticity (arising from inherent randomness in demographic processes of individuals) and corresponding extinction risk (Conner & White 1999; Jager, 2001; Kendall & Fox 2002; Vindenes *et al*. 2008). We are not aware of studies considering consequences for any of the other demographic outputs mentioned above.

Individual heterogeneity can arise from additive genetic inheritance in fitness-related traits. Such traits can be quantitative genetic traits such as height or body mass, or discrete traits determined by few alleles (Coulson *et al*. 2011). Because they apply to continuous traits, IPMs have recently been proposed as a useful tool to model eco-evolutionary dynamics (Coulson *et al*. 2010; Smallegange & Coulson 2013). As they belong to the same general model class as matrix models they have the same analytical advantages (Ellner & Rees 2007). However, so far a majority of applications have been based on one trait (usually some measure of body size), assuming all individuals have the same life history. Individual heterogeneity is sometimes accounted for in the estimation of parameters through random effects in the intercepts and/or slopes (Ellner & Rees 2007; Coulson, 2012), but the final IPM is usually constructed for the mean life history, omitting these effects. Moreover, individual-level properties such as genotypes can affect the life history in complex ways that are difficult to capture through random effects. In a model based on just one trait, it can also be challenging to define heritability, in particular if the focal trait is one that develops over the lifetime (such as size). The so-called age–stage-structured models (Coulson *et al*. 2010) resolve some of these issues, but require one heritability measure for each age, and individual traits are not explicitly included.

Here, we propose a conceptual demographic framework including individual heterogeneity in life histories, that can apply to both continuous (quantitative genetic) and discrete traits. We define the model in a general way that allows for several mechanisms of inheritance to be considered, both genetic and non-genetic (Danchin, 2013). This framework represents a complementary approach to age–stage-structured models that include individual-specific traits and that only requires one heritability estimate, rather than one for each age. We apply the framework to evaluate consequences of ignoring heterogeneity for a range of population dynamical and life-history parameters, extending and synthesising our knowledge of such effects. Second, we discuss how the framework can help advance demographic approaches to model eco-evolutionary dynamics and population responses to environmental impacts.

## Conceptual Model Framework

**Figure 1:**
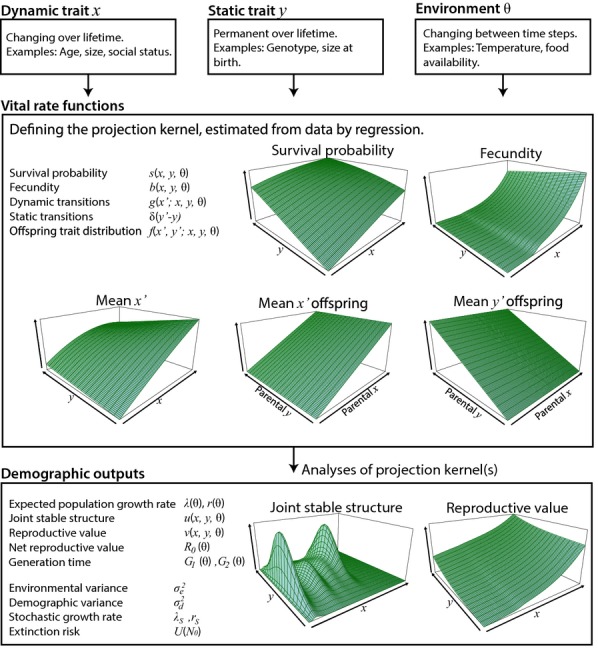
A schematic overview of the main model components and the demographic outputs considered in the analyses. Together with environmental variable(s) *θ*, the dynamic trait *x* and static trait *y* define individual vital rate functions. Here, these are illustrated for a constant environment (only means are shown for the offspring trait distribution and the distribution of dynamic transitions). The offspring inheritance is a joint distribution for 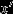 and 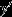, in this illustration they are independent. Once vital rates are defined, demographic outputs are obtained by analysis of the projection kernel, for instance the stable structure and reproductive value in a given environment, as shown here. The final four outputs require an extension of the model to include demographic and/or environmental stochasticity (Appendix S1).

The framework is based on IPMs for continuous traits and matrix models for discrete traits (and can include combinations of the two). For simplicity of notation we will present the model and results within the IPM framework, but all results are easily converted to the case of discrete traits, replacing integrals by sums. A schematic overview of the framework and the demographic outputs considered is given in Fig. 1, and a more detailed model description is given in Appendix S1. Calculations were done using the software R (R Development Core Team, 2013), and R code for all examples is provided as supplementary material.

**Box 1 General description of integral projection models**

Integral projection models (IPMs) were introduced for size-structured populations (Easterling *et al*. 2000), and are the continuous-state analogue to matrix models. Both matrix models and IPMs belong to the same general model class of discrete time steps, and an IPM can be thought of as a high dimension matrix model. In the simplest case of an IPM, the population is structured according to one trait (e.g. size) *x*, with a sample space 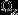 (e.g. the range from 0 to infinity in the case of size). In contrast to most matrix models, vital rates of IPMs are estimated from data using regression techniques. There is an implicit assumption that the structuring trait carries information that can be used to predict the vital rates. Two recent papers describe general methods for construction and analysis of IPMs from data, including numerical methods for calculation and model diagnosis (Merow *et al*. 2014; Rees *et al*. 2014).

The individual level processes of survival, reproduction and transition between trait values are characterised by four main vital rate functions: (1) survival probability *s*(*x*), (2) a distribution 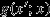 describing transition in trait value from *x* to 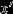, conditional on survival, (3) fecundity *b*(*x*) and (4) a distribution 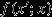 for offspring trait *x*′ given parental trait *x*. Together, these functions define the projection kernel,

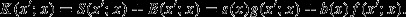

The projection kernel describes the contribution from individuals in state *x* to state 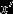 the next time step, in terms of survival/trait transitions and reproduction/offspring trait allocations. Letting *n*(*x*) describe the population density of individuals across the trait *x*, the total population size is 
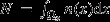
. The population size the next time step is given by 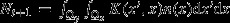. By analysis of 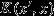 we find the parameters describing asymptotic properties, i.e. the long-term growth rate *λ* (average fitness), the stable structure *u*(*x*) and reproductive values *v*(*x*) (Easterling *et al*. 2000). The functions *u*(*x*) and *v*(*x*) also define the sensitivity surface of *λ* to the projection kernel (Ellner & Rees 2006).

IPMs have been applied on a number of organisms from different taxa, with various extensions such as age structure, spatial structure, environmental drivers, density dependence, species interactions and demographic and environmental stochasticity (see summary and references in Rees *et al*. 2014).

### Static and dynamic traits

Demographic models are often based on one or a few traits, typically age in matrix models, or body size in IPMs. To include individual heterogeneity in life histories, we propose a framework including two types of traits, classified as static and dynamic (Hill *et al*. 1999). Static traits remain the same over the lifetime of an individual, or represent events that occur only once and have a lasting impact on the individual life history. Such traits can therefore capture individual effects beyond the population level average. Examples of static traits could be body size at birth, genotype or spatial location of a sessile organism. Dynamic traits are changing over the lifetime, randomly or non-randomly. Age and body size are particularly important cases of dynamic traits, as either one or both are often found to be a major determinant of vital rates (Caswell, 2001). Other examples that may be important for some organisms include spatial location (mobile organisms), body condition, or social status. A non-comprehensive list of empirical studies including various types of static and dynamic traits is provided in Table 1. Among these, we see that the static trait is often some property of the environment, while the dynamic trait is often age, life-history stage or some measure of organism size.

**Table 1:** Examples of empirical studies including static and dynamic traits (not a comprehensive overview), including the type of traits considered and the vital rates found to be affected by the static trait in the study species (not including theoretical model explorations). In most studies, the dynamic trait was found to affect all vital rates

Species	Dynamic trait	Static trait	Vital rates affected by static trait	References
Pike (*Esox lucius*)	Body length	Length at age 1	Growth	This study, Vindenes *et al*. (2014)
Red-billed chough (*Pyrrhocorax pyrrhocorax*)	Age	Natal habitat	Survival	Reid *et al*. (2004, 2006)
Eurasian oystercatcher (*Haematopus ostralegus*)	Life-history stage	Natal habitat	Survival, fecundity	van de Pol *et al*. (2006, 2010)
Great tit (*Parus major*)	Age	Personality (behaviour in new environment)	Survival, fecundity	Dingemanse *et al*. (2004)
Grey wolf (*Canis lupus*)	Body weight	Genotype (coat colour)	Survival, fecundity	Coulson *et al*. (2011)
Columbian ground squirrel (*Urocitellus columbianus*)	Body weight	Sex	Fecundity (mating function)	Schindler *et al*. (2013)
Red deer (*Cervus elaphus*)	Age class	Environmental and density conditions in birth year	Male survival, fecundity	Rose *et al*. (1998)
Roe deer (*Capreolus capreolus*)	Body weight, age class	Birth date	Growth, survival	Plard *et al*. (2015)
Brook trout (*Salvelinus fontinalis*)	Age	Habitat (food)	Survival, growth, fecundity	Hutchings (1992)
Coho salmon (*Oncorhynchus kisutch*) Chinook salmon (*O. tshawytscha*)	Age	Natal habitat (freshwater)	Male fecundity, growth (maturation decision)	Vøllestad *et al*. (2011)
Bulb mite (*Rhizoglyphus robini*)	Body length	Food type (habitat)	Survival, growth, fecundity	Smallegange *et al*. (2014)
Lady orchid (*Orchis purpurea*)	Total leaf area	Habitat (light conditions)	Survival, fecundity	Jacquemyn *et al*. (2010)
Tree cholla cactus (*Opuntia imbricata*)	Plant volume	Elevation (herbivory level)	Fecundity, growth	Miller *et al*. (2009)
White hellebore (*Veratrum album*)	Stem diameter	Habitat type	Survival, growth, fecundity	Hesse *et al*. (2008)

Static traits will often result from conditions of early development, a particularly important time in the life cycle of most organisms (Lindström, 1999; Beckerman *et al*. 2002; Cam *et al*. 2003; Monaghan, 2008). For instance, in early ontogenetic stages, nutritional conditions can have lasting effects on development and growth (Metcalfe & Monaghan 2001). Maternal effects and cohort effects are general concepts characterising such early effects, and both are commonly found in natural populations (Beckerman *et al*. 2002). Maternal effects represent parental influences beyond additive genetic effects (Mousseau & Fox 1998), while cohort effects refer to lasting effects of environmental factors (Beckerman *et al*. 2002). Moreover, in many organisms, the early stages of the life history often correspond to the time when individuals are most likely to migrate or disperse to new environments. Sessile organisms generally follow such a life history, but even in some mobile animals adults may show more limited dispersal than juveniles, for instance after they establish a territory.

In a population model based on Markov processes, Tuljapurkar *et al*. (2009) distinguished between dynamic and fixed heterogeneity in life-history trajectories. In that model dynamic heterogeneity referred to stochastic transitions between stages, while fixed heterogeneity referred to unobserved or measured differences generating random variation in life histories. These concepts are similar to our distinction between dynamic and static traits, but not quite the same since fixed vs. dynamic heterogeneity focus on classifying variability in stochastic processes. Here, we use the terms static and dynamic to classify the traits that structure the population.

### Vital rates and projection kernel

The vital rates are one way to summarise the life history of an organism (Caswell, 2001). In IPMs, these are functions describing how survival, reproduction and trait development depend on the static and dynamic traits and the environment. For the sake of simplicity, we will consider cases with only one static and one dynamic trait, but note that in general the framework can include more than one trait of each type. Let *x* and *y* denote the dynamic and static trait, respectively, and let the environment be described by a variable *θ* representing one or more environmental variables like temperature, rainfall or resource availability, taking a new value each time step. For simpler notation, we will omit *θ* where suitable. Interactions between *x*, *y* and *θ* over time will shape the life history (i.e. vital rates) of each individual, including mechanisms of inheritance (Fig. 1). Each individual will have a constant value of *y* set at birth, but will experience different values of *x* and *θ* over the lifetime. Together, the effects of the static and dynamic traits on the vital rates can be combined in a number of ways, including interactions between them, so that a range of different life histories can be modelled using only two traits.

The population density distribution describing the expected number of individuals across the two traits is given by the function *n*(*x*,*y*). Changes in this joint distribution over time is described by the projection kernel, which is a function of all the underlying vital rate functions (Easterling *et al*. 2000). For an IPM of static and dynamic traits, it is given by


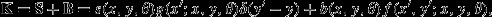
(1)

where the survival kernel **S** describes survival and transitions of the dynamic trait, while the reproduction kernel **B** describes production of offspring including the offspring distribution of both traits. Two important aspects separate this model from a model defined for only one trait: (1) the Dirac delta function 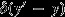 enters the survival kernel to keep the static trait *y* constant for an individual during its lifetime (in the case of a discrete trait matrix model this would be replaced by a Kronecker delta), and (2) in the reproduction kernel, the joint distribution 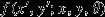 allows the static and the dynamic trait to be correlated at birth (or whenever offspring are counted), which is likely often the case. For example, if the static trait is within-season timing of birth and the dynamic trait is body size, then early born individuals will have more time to grow so that if offspring are counted as 1-year-olds the two traits will be correlated at this point.

The joint distribution 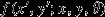 allows for many mechanisms of inheritance to be considered (Danchin, 2013), including (1) no inheritance, i.e. no effect of parental traits, (2) cohort effects through the environment *θ*, (3) additive genetic inheritance of the static trait *y* and (4) maternal effects (including epigenetic inheritance) through the dynamic trait *x* and interactions with *y* and/or *θ*. The specific form of the offspring trait distribution 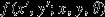 will in each case depend on the relevant mechanisms for inheritance, that may lead to a frequency-dependent model. The model definition here is general, and for each specific application, the potential mechanisms of inheritance should be considered. For instance, the general definition does not preclude mechanisms causing runaway selection (Darwinian demons). This could occur, for instance with directional selection on a trait where offspring values do not regress towards a mean.

### Demographic outputs calculated from the model

Several demographic outputs can be calculated based on the projection kernel or projection matrix (methods are essentially the same for matrix models and IPMs; Easterling *et al*. 2000; Caswell, 2001; Ellner & Rees 2006). In particular, the dominant eigenvalue corresponds to the long-term population growth rate *λ*, while the right and left eigenvectors correspond to the joint stable trait structure **u** = *u*(*x*,*y*) (scaled so that 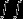
*u*(*x*,*y*)d*x*d*y* = 1) and reproductive values **v** = *v*(*x*,*y*) (scaled so that 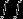
*u*(*x*,*y*)*v*(*x*,*y*)d*x*d*y* = 1), respectively (Caswell, 2001; Ellner & Rees 2006). The marginal distributions of *x* and *y* are found by integrating *u*(*x*,*y*) with respect to the other trait. The stable structure and reproductive values also define the sensitivity (and elasticity) of *λ* to the projection kernel (Ellner & Rees 2006).

Other widely applied demographic outputs are the net reproductive rate and generation time. The net reproductive rate 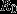 describes the expected number of offspring over the lifetime. It is a measure of the level of reproductive output as well as the generation-to-generation population growth rate, while the generation time measures the timing of reproduction and life-history speed (Steiner *et al*. 2014). For structured models with overlapping generations, different measures of generation time are available (Caswell, 2001). Here, we will consider two of them, the time it takes for the population to grow by a factor 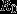, 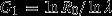 (Caswell, 2001), and the mean age of mothers in a population at the stable distribution, 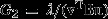 (Bienvenu *et al*. 2013).

So far, we have considered properties of a deterministic model in a constant environment. The model can also be extended to stochastic dynamics arising from demographic and environmental stochasticity (Vindenes *et al*. 2012). Demographic stochasticity arises from inherent randomness in the processes of survival, reproduction and transitions of traits, while environmental stochasticity arises from fluctuations in the environment affecting the underlying vital rates (May, 1973). The demographic and environmental variance are parameters that measure the amount of each type of stochasticity in the population growth (Engen *et al*. 2009; Vindenes *et al*. 2012). In general, estimating the long-term properties of stochastic dynamics in structured populations is complicated by transient fluctuations in the trait distribution leading to temporal autocorrelation in the population size. Engen *et al*. (2007) demonstrated for an age-structured model that the dynamics could be described by considering the total reproductive value, calculated using the reproductive values of the mean environment that work as a filter for the transient fluctuations. This method was later generalised to stage-structured cases, both for IPMs and matrix models (Vindenes *et al*. 2012).

## Applications

### Consequences of ignoring heterogeneity

For most natural populations, we will never know all sources of heterogeneity, or be able to include them all in a model. Even if this were possible, obtaining the required data can be costly and time consuming, and the extra effort may not necessarily lead to a large difference in the results of interest. Thus, it is important to evaluate the potential consequences of ignoring heterogeneity, not the least whether certain parameters are more robust than others. In this section, we describe a general method for how this can be done, both for the case where the heterogeneity is completely ignored and the case where it is only partly ignored (that is, ignoring the static trait *y* and/or the dynamic trait *x*). In Appendix S2, we derive results for a range of demographic outputs, and a summary is provided in Table 2.

**Table 2:** Summary of demographic outputs in models ignoring all or part of the underlying heterogeneity in a population (here assumed to be defined by two traits), calculated in Appendix S2

Parameter	Heterogeneous model, structured in *x* and *y*	Partial heterogeneity (model ignoring *y*)	No heterogeneity (model ignoring *x* and *y*)
Stable distribution	**u** = *u*(*x*,*y*) (right eigenfunction of **K**)	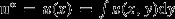 (right eigenfunction of 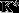 )	1
Density distribution, population size	**n** = *n*(*x*,*y*), *N* = 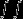 *n*(*x*,*y*)d*x*d*y*	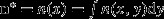 , *N* = 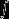 *n*(*x*)d*x*	*N* = 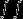 *n*(*x*,*y*)d*x*d*y*
*Mean vital rates*			
Survival	*s*(*x*,*y*)	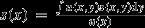	*s* = 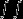 *u*(*x*,*y*)*s*(*x*,*y*)d*y*d*x*
Fecundity	*b*(*x*,*y*)	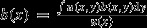	*b* = 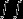 *u*(*x*,*y*)*b*(*x*,*y*)d*y*d*x*
Transitions in *x*	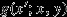	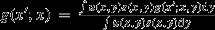	1
Offspring distribution *x*	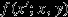	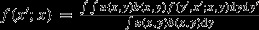	1
*Reproductive value moments*			
Reproductive value, total RV	**v** = *v*(*x*,*y*), *V* = 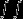 *v*(*x*,*y*)*n*(*x*,*y*)d*y*d*x*	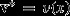 , 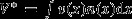	1,*N*
Mean after survival	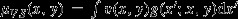	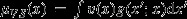	1
Mean for offspring	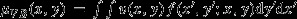	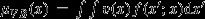	1
Variance after survival	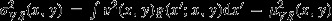	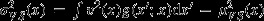	0
Variance for offspring	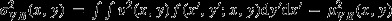	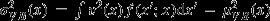	0
*Demographic stochasticity*			
Variance survival	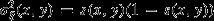	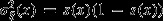	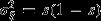
Variance fecundity	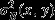	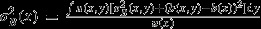	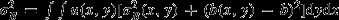
Covariance survival/fecundity	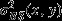	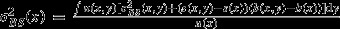	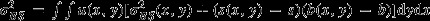
*Kernels*			
Projection kernel	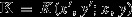	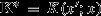	*s* + *b*
Survival/transition kernel	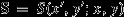	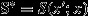	*s*
Reproduction kernel	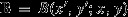	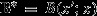	*b*
Net reproduction kernel	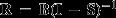	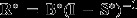	b/(1 - s)
*Demographic outputs*			
Mean growth rate	*λ* = Dominant eigenvalue of **K**	*λ* = Dominant eigenvalue of 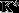	*λ* = *s* + *b*
Net reproductive rate	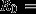 Dominant eigenvalue of **R**	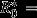 Dominant eigenvalue of 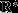	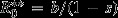
Generation time 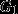	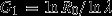	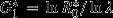	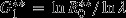
Generation time 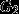	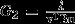	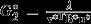	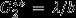
Demographic variance	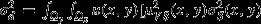 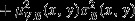 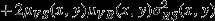 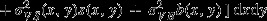	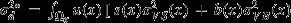 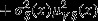 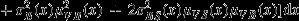	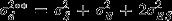
Environmental variance	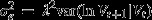	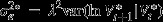	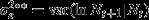
Stochastic growth rate	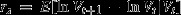	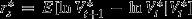	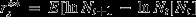
Extinction risk at *N* = 1	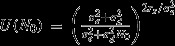	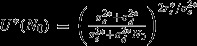	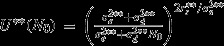

The heterogeneous model has structure due to a static trait *y* and a dynamic trait *x*. One model ignores variation in *y* and the other model ignores both *x* and *y*. All integrals are taken over the entire range of the trait in question. All functions generally also depend on the environment *θ* (not included for more concise notation). The full equation for the demographic variance is provided in Appendix S1.

The term ‘heterogeneity’ has been used differently in the biological literature, which is important to keep in mind. In many cases, for instance studies on statistical estimation of vital rates (e.g. Burnham & Rexstad 1993), heterogeneity is implicitly assumed to mean hidden and unobserved individual differences. This is also the interpretation in studies of effects of heterogeneity on patterns of ageing (Vaupel *et al*. 1979). When the goal is to evaluate consequences of ignoring such hidden heterogeneity, the problem is one of modelling error, as the underlying population is the same. Other studies consider effects of changing heterogeneity in the underlying population, for instance on the distribution of lifetime reproductive success (Tuljapurkar *et al*. 2009; Caswell, 2011) and on the long-term population growth rate (Kendall *et al*. 2011). This problem is one of comparing different underlying populations rather than models.

Both of these general questions are interesting, but it is important to be aware of their differences, in particular when results of different studies are compared. For instance, Kendall *et al*. (2011) found that heterogeneity affects the expected growth rate *λ*, comparing a population where vital rates change over age with a uniform population starting with the same vital rates at birth that remain constant over age. Since these populations are different, this study did not evaluate consequences of ignoring heterogeneity, but rather effects of specific changes in the underlying population. Based on this result Kendall *et al*. (2011) suggested that failure to recognise this effect on *λ* had affected results of other studies, including a study of Vindenes *et al*. (2008) on demographic stochasticity. However, the focus of Vindenes *et al*. (2008) was to evaluate consequences of ignoring heterogeneity (comparing models, not populations) so there was by definition no effect on *λ* in their model (a known demographic result that we demonstrate analytically for this model in Appendix S2). In the following, we will consider effects of ignoring heterogeneity, but in general the presented framework can also be used to explore consequences of changes in the underlying population.

### General approach

To evaluate the potential consequences of ignoring heterogeneity, we compare demographic outputs calculated from a model including heterogeneity (in this case, defined by a model with a static and a dynamic trait) to the corresponding parameters from models that partly or completely ignores the heterogeneity, i.e. ignoring either the static trait or both the static and dynamic trait. To derive the vital rates of these comparison models, we use the stable trait distribution (see details in Appendix S2). The model where both traits are ignored corresponds to an unstructured model of a uniform population, in which only the estimates of survival and fecundity are required to describe the dynamics. The case where only the static trait is ignored is slightly more complex, because transition functions of the dynamic trait must be derived as well. Table 2 summarises the resulting expressions for the vital rates for the underlying heterogeneous model and the two comparison models, as well as the resulting expressions for all the demographic outputs considered. A main conclusion from this analysis is that except for *λ*, all parameters are generally affected by ignoring heterogeneity.

The amount and direction of the bias in the models ignoring heterogeneity depend on the particular case being considered, that is the general life history, which vital rates are impacted by heterogeneity, and the amount of heterogeneity. While it is difficult to make predictions on the direction and amount of bias for the general case, the methods and results presented here provide a useful tool for evaluating consequences of heterogeneity in specific situations (to which the supplemented R code should be easily adapted). Two examples are provided below, one theoretical and one empirical, where some of these issues are discussed in more detail.

#### Example 1 Red and green size-structured population:

**Figure 2:**
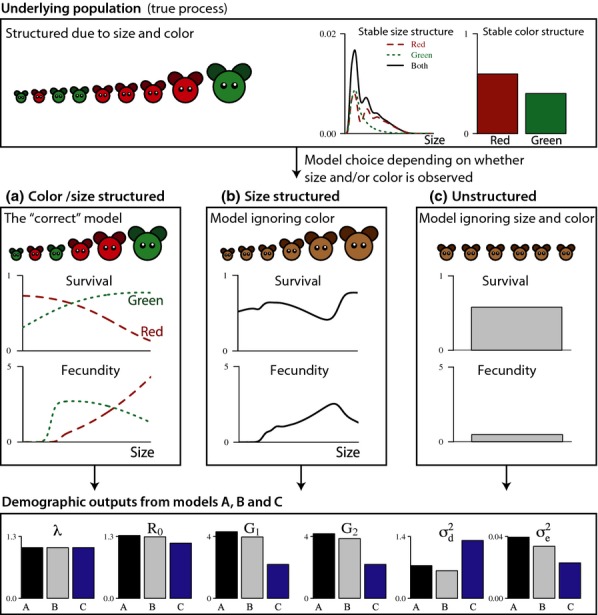
An example of a size-structured population of red and green individuals. Depending on whether colour and size is recognised, the vital rates will look different to the observer, as illustrated for survival and fecundity in panels (a–c) (for transition rates, see the provided R code). As a result, with the exception of *λ* estimates of demographic outputs will be biased in models b and c. Consequences of underlying heterogeneity on estimates of extinction risk (through demographic and environmental variance) are provided in the supplementary R code.

To illustrate the results for a simple case where the ‘true’ population structure is known, we developed a theoretical example of a size-structured population with red and green individuals (Fig. 2, supplementary R code). Here, each vital rate depends on size as well as colour, and each colour represents a different life-history strategy. For the purpose of illustration, we assume that this population is studied by three biologists, where the first recognises both size and colour and includes them in the model. The second biologist is colour-blind (incidentally, this is the case for one of the authors of this study) and only includes the size differences. The third is in a hurry and does not account for either size or colour, modelling all individuals as equal. How different would their conclusions on the various demographic outputs be, assuming there are no other sources of error than the model choice?

Figure 2 compares the demographic outputs obtained from each case. With the exception of *λ*, all parameters are biased in the models ignoring colour and/or size, and the model ignoring both traits is generally doing the worst job, as expected. The demographic variance stands out from the general pattern, as the model ignoring only colour underestimates it while the unstructured model overestimates it.

#### Example 2 Consequences of early growth differences in pike:

As an empirical example, we consider the demography and dynamics of a population of pike (*Esox lucius*) from Windermere, UK, for which unique long-term individual-based data are available (Le Cren, 2001). A length- and temperature-dependent IPM was recently developed for this population (Vindenes *et al*. 2014). In Appendix S3, we extend this model to also include individual effects through length at age 1 (*y*, static trait), reflecting early growth differences. Note that in this example, the static and dynamic traits are the same (and therefore perfectly correlated) in offspring. The exact underlying mechanisms of early growth differences are not known, but likely arise from a combination of genetic and environmental effects. From other studies, we know that temperature and food availability during the first year are key determinants of somatic growth (Casselman, 1996).

**Figure 3:**
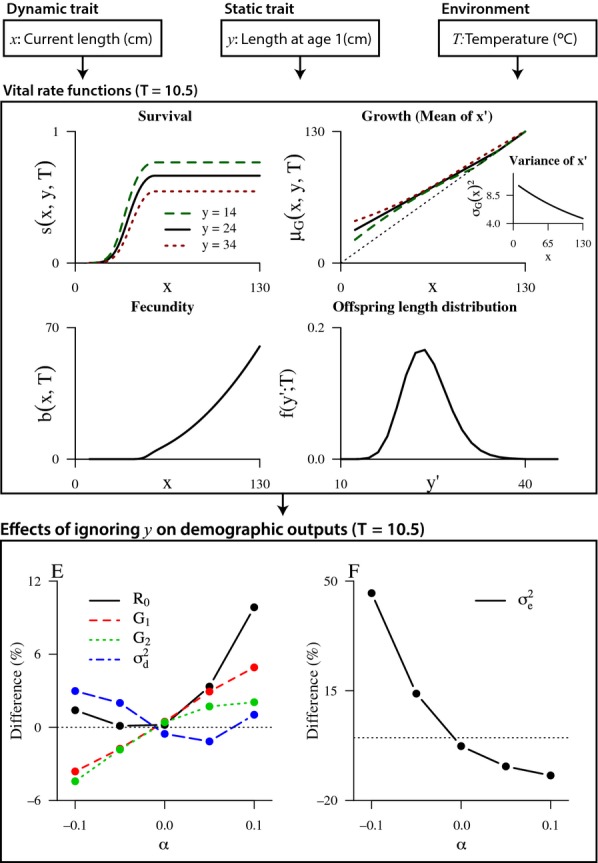
A length- and temperature-based model for pike, including length at age 1 as a static trait *y* in addition to length *x* and temperature *T*. Vital rates (A–D) are shown here for the mean temperature (in this example 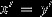 for offspring), for values in other temperatures see Appendix S3. The parameter *α* measures the effect of *y* on survival, negative values correspond to a negative effect and thus a trade-off with growth. Positive values correspond to positive effects representing ‘quality’ differences among individuals. Panels (E and F) show the resulting bias in various demographic outputs in a model that ignores *y*, as a function of *α*.

Figure 3 shows the vital rates as functions of the static and dynamic trait. Size ranks among individuals are largely maintained over the lifetime, so that initially large pike tend to grow persistently faster over their lifetime and reach a larger final size than those starting out small. These growth differences lead to lifelong fitness consequences through the indirect effects of *y* on survival and fecundity, as larger individuals tend to have higher values of these rates (Fig. 3). In addition, we included a direct effect of *y* on survival, measured by the constant *α* (Appendix S3). Negative values of *α* correspond to a survival trade-off associated with rapid early growth, while positive values correspond to a case where rapid growth is also associated with higher survival (‘quality’ differences). The data were not sufficient to quantify this effect, but indicated it would be negative (Appendix S3). We therefore evaluated the results for a range of values of *α*.

The results show that the consequences of ignoring heterogeneity in *y* (Fig. 3) are smallest when *y* only affects growth (*α* = 0). The growth model ignoring heterogeneity was in this case able to capture the average growth quite accurately, and the main error arising from ignoring heterogeneity is that the systematic individual variation is treated as random variation, with limited consequences for most demographic outputs. In addition, since *y* is approximately lognormally distributed, the amount of heterogeneity is not extremely large in this model. However, when *y* is also affecting survival, either through a positive or a negative effect, the consequences of ignoring heterogeneity become more severe for all the demographic outputs (Fig. 3).

This example demonstrates the potential importance of life-history trade-offs also for the consequences of ignoring heterogeneity. Although life-history theory suggests that trade-offs between vital rates should be common (Roff, 1996), identifying them in empirical systems can be difficult (van Noordwijk & de Jong 1986). A potential explanation could be that individual differences in resource acquisition affect the expression of the trade-offs so that they are stronger for individuals with low resources (Reznick *et al*. 2000). Here, the initially small pike increases their growth rate much at first, almost catching up with the larger ones, but then the growth rate declines and they tend to stop growing at a smaller final size (Fig. 3). This pattern could potentially result from a stronger trade-off between growth and fecundity in these small individuals (being expressed when they start reproducing).

### Eco-evolutionary dynamics

In this section, we discuss another potential application of the conceptual model framework, to study eco-evolutionary dynamics. It is now widely recognised that evolutionary changes can happen also over shorter time scales that are relevant to ecological processes (Schoener, 2011). Ecological and evolutionary processes interact to determine the dynamics of a population, through simultaneous changes in the underlying genetic and demographic structure (Pelletier *et al*. 2009; Smallegange & Coulson 2013). Thus, both processes should be considered when aiming to understand and predict population responses to external impacts, such as harvesting or climate change. The topic of eco-evolutionary dynamics is wide, and a number of different approaches are available, some of which also consider structured populations (Hairston *et al*. 2005; Pelletier *et al*. 2009; Ellner *et al*. 2011; Schoener, 2011). Here, we focus on demographic approaches (IPMs and matrix models) and how the model framework introduced here can provide a useful step towards more realistic descriptions of eco-evolutionary dynamics in these models.

The term eco-evolutionary dynamics has also been used differently in the literature. Smallegange & Coulson (2013) define it as simultaneous changes in parameters of interest to ecologists, such as population size, and in parameters of interest to evolutionary biologists, such as strength of selection. Others have used more restrictive definitions requiring a change in the genetic composition in response to selection as well as in population dynamical properties (Pelletier *et al*. 2009; Schoener, 2011). Studying simultaneous responses in multiple life-history parameters, as advocated by Smallegange & Coulson (2013), provides a more detailed picture than the responses of *λ* (average fitness) alone. But demographic models such as matrix models have been applied to study such simultaneous changes for a long time without using the term eco-evolutionary dynamics (Caswell, 2001). Changes can occur in parameters that are *important* to evolution (e.g. age-specific selection differentials), without necessarily leading to an evolutionary change in the underlying genetic composition (which also requires available genetic variation for selection to act upon). To emphasise this point, we therefore prefer the more narrow definition assuming changes in the genetic composition, even though it can be challenging to detect such changes empirically. Note that this definition does not preclude models or analyses based on the phenotypic level.

Since IPMs incorporate continuous traits as well as potential heritability (i.e. part of the variation in a static trait that is genetically transmitted to offspring; Danchin, 2013) through the offspring trait distribution, they provide a good starting point for modelling eco-evolutionary dynamics (Smallegange & Coulson 2013). However, so far many applications have been based on one trait, usually a measure of body size (but see e.g. Plard *et al*. 2015). Taking body size as an example, we highlight three limitations to such a model in describing evolutionary dynamics. First, if heritability is modelled through a positive correlation between parent and offspring size, then genetic effects are confounded with ontogeny since individuals tend to grow over life. Then, older parents will also tend to have larger offspring than younger parents, regardless of their own size as offspring. Second, initial differences in offspring size will tend to become weaker over age in such a model, since latent individual differences in growth will be included as random variation instead. Thus, if somatic growth has a genetic component causing some individuals to grow consistently faster than others, this would not be sufficiently captured by a model based on size alone. Third, any potential non-linear effects of genotype (static trait) on growth rate as well as on other vital rates, are ignored, as are potential correlations between vital rates (such as trade-offs) induced by the genotype. These issues apply to all models that include only a dynamic trait and aim to consider eco-evolutionary dynamics, but can largely be mitigated by including a static trait representing individual effects.

In an interesting recent study of hunted bighorn sheep (*Ovis canadensis*) Traill *et al*. (2014) used a size-structured two-sex IPM to evaluate the causes of changes in the body size distribution, including heritability of body size. The results of this model suggested that effects of heritability were small compared to other causes, i.e. that evolution was less important than demography. Our model framework could be applied to this system, for instance using offspring size as a static trait (as for pike in the above model). This would provide a robustness check of these results by separating genetic effects from ontogenetic development, and by allowing for lasting individual effects of offspring size through the life history.

Both dynamic and static traits will in most applications to natural populations be phenotypic traits, as the underlying genetics are typically not observed. Continuous traits can often be treated as quantitative genetic traits (assumed to be determined by many genes of small effect), decomposing variation into an additive genetic and an environmental component (Coulson *et al*. 2010). Heritability should ideally be estimated from genetic data, but could also be estimated from the slope of the parent–offspring regression of observed phenotypes (Coulson *et al*. 2010). Discrete traits are sometimes determined by just a few alleles on one locus, and in a few cases the underlying mechanisms of genetic inheritance can be explicitly modelled. The wolf model employed by Coulson *et al*. (2011) provides a nice example, where coat colour (black or grey) is determined by two alleles on a locus. In Appendix S4, we provide another example based on the model of red and green individuals in example 1. In these cases, the dynamics of the model become frequency dependent, and will also depend on assumptions regarding the mating system.

Another demographic approach to modelling eco-evolutionary dynamics is that of age–stage-structured models (Coulson & Tuljapurkar 2008). This is a flexible framework where individual variation in life histories can be included through various combinations of ages and stages. The model with static and dynamic traits can in principle be converted to an age–stage-structured model, as individuals always have an age, regardless of whether it is measured (Caswell, 2001). It is conceptually different by focusing on separating dynamic and static traits (note that the dynamic trait can be age, but is not limited to this), and the static trait is always constant over the lifetime of an individual. In age–stage-structured models, age is always included, and the stage may also change over the lifetime (e.g. size). The frameworks also differ in how heritability is included. Because the static trait is constant over the lifetime, only one heritability estimate is required for this framework (i.e. one for each static trait) as genetic effects can be modelled in this trait. An age–stage-structured model generally requires one heritability estimate for each age. The two frameworks, age–stage-structured models and the framework discussed here, thus represent different and complementary ways of thinking about eco-evolutionary dynamics in structured populations. We believe that the new perspective provided by separating static and dynamic traits will be a useful addition to the expanding toolkit of demographic models, in particular for applications to eco-evolutionary dynamics.

#### Example 3 Effects of temperature on demographic outputs in pike:

**Figure 4:**
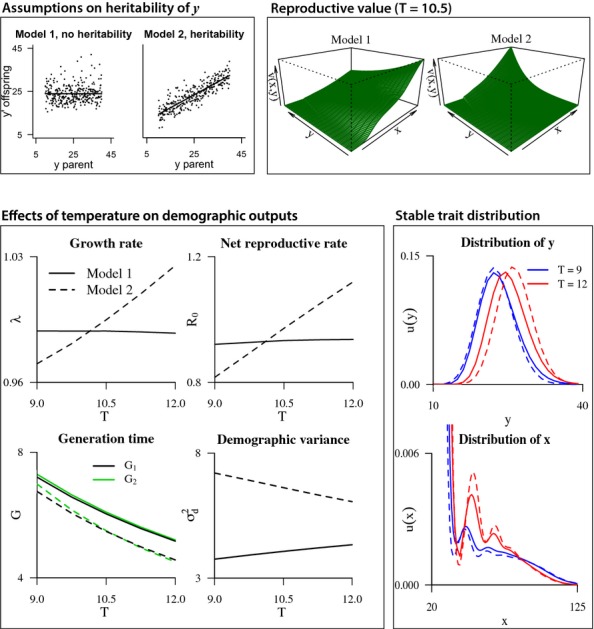
An example of eco-evolutionary dynamics in the pike model including length at age 1 as a static trait *y*, in addition to length *x* and temperature *T* (details in Appendix S3). A model with zero heritability of *y* (model 1) is compared to a model with a heritability of 0.6 (model 2), as shown in the upper left panels. The resulting reproductive value functions for the two models are shown for the mean temperature. The lower left panel shows effects of temperature on various demographic outputs in the two models, while the lower right panel shows the marginal stable trait distributions of *y* and *x* for two temperatures, in the two models.

As an example of how conclusions can be affected by changing heritability of the static trait, we again consider the pike model introduced in the previous section (example 2). Here, the static trait *y* is length at age 1 and the dynamic trait *x* is length. In addition, the model includes temperature effects on each vital rate (Appendix S3; Vindenes *et al*. 2014). We now ask how the various demographic outputs depend on temperature, and compare the results for two scenarios: one with zero heritability of *y* (as in example 2), and one with a heritability of 0.6 (slope of relationship between parent and offspring values of *y*; Fig. 4). Note that this heritability is not estimated from data, as we do not have the pedigree, and is here assumed to represent additive genetic effects. The model is female-only, thus we implicitly assume that males and females have the same life history in this example. In the model with a 0.6 heritability, the intercept of the mean offspring length was adjusted and variance in offspring length was reduced, so that the offspring length distribution would be the same for the two scenarios in the mean environment (temperature 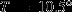
C). In this example, we assume a negative effect of *y* on survival (*α* = −0.05) in addition to the estimated effects on somatic growth as shown in Fig. 3.

To evaluate the relative individual advantage of a large or small *y*, we considered the reproductive value, measuring the (expected) contribution of an individual with a certain trait combination to future population growth, relative to other individuals (Fig. 4). Note that this is not a measure of fitness in the sense that it predicts the increase of individuals of a certain *y* value (having a genetic component) relative to others, as for a given environment (temperature) all trait combinations will eventually grow with the same rate *λ* (Caswell, 2001). However, it does tell us which individuals and trait combinations are the main drivers of the population dynamics within each environment and heritability scenario. In the model including heritability, individuals of large *y* (and *x*) have the highest reproductive value, while the pattern is opposite when the heritability of *y* is zero (shown in Fig. 4 for the average temperature; these patterns are not qualitatively affected by temperature changes).

The results (Fig. 4) also revealed a large effect of heritability on the temperature effect on all demographic outputs, even reversing the effect in many cases. The underlying mechanisms are complex because temperature has different effects on each vital rate across body length. In short, with no heritability of *y* any effect of this trait on fitness occurs through survival and growth. Since the reproductive value is smaller for individuals of large *y* in this case, the positive effects of increased somatic growth do not outweigh the negative effects on later survival. In the model including heritability, however, *y* can also affect fitness through reproduction. Here, *y* will have a positive effect through offspring length since larger offspring tend to have a higher survival than smaller ones. Together, the positive effects of *y* on reproduction (through offspring size) and growth are in this case outweighing the negative effects on survival. This example highlights the potentially large role of heritability in eco-evolutionary dynamics.

Finally, we considered the changes in the stable trait distribution of *x* and *y* between two temperatures (cold and warm), for the two heritability scenarios (Fig. 4). In both cases, the distributions are changing with temperature, demonstrating that even in a model without heritability the trait distribution will shift. However, the effects are larger for the model including heritability, as in this case the net effect of temperature works in the same direction as the response to selection on body size (because large *y* individuals are at an advantage in the model including inheritance, the stable distribution is shifted towards higher mean *y*). In other cases, however, it is possible at least over shorter time periods that environmental plastic changes acts in opposite direction to the effects of selection, resulting in an apparently unchanging phenotypic trait distribution even if the genotypic composition is altered (Hairston *et al*. 2005).

## Discussion

Individual heterogeneity in life histories plays an important role in both ecological and evolutionary processes, but can be challenging to incorporate in models (Metcalf & Pavard 2007; Smallegange & Coulson 2013). We have introduced a conceptual framework of IPMs/matrix models separating static and dynamic traits, providing a flexible model for effects of heterogeneity. We now discuss some challenges and future opportunities for applications of such a framework.

The framework represents a complementary approach to age–stage-structured models, where individual effects are more explicitly included through the static trait. Instead of measuring a heritability at each age, only one measure is required. In the parameterisation of IPMs, individual heterogeneity is often accounted for in the statistical estimation of vital rates, by including individual random effects in mixed models (Ellner & Rees 2007; Coulson, 2012). Using the framework presented here, the random intercept (or slopes) from such regressions could be used to define a static trait representing individual ‘quality’, an approach that could be particularly useful when the cause of individual heterogeneity is unknown (note that this approach can also be used to expand age/stage structured models to include individual heterogeneity). However, potential non-linear effects of the individual trait on the vital rates as well as interactions with the dynamic trait and environmental variables are then more difficult to include.

By separating static and dynamic traits effects of ontogeny can be separated from genetic effects in IPMs. It is likely that effects of heritability then become stronger than in IPMs based on just one (dynamic) trait. For instance, in our pike example, we found strong potential effects of heritability on the estimated population responses to temperature (example 3, Fig. 4). The study of Traill *et al*. (2014) based on a model with one dynamic trait did not find a strong effect, but applies to a species with a very different life history than pike. Future applications of the framework presented here could explore in more detail under which circumstances evolution is expected to explain more of the observed phenotypic changes, and for which species. It can also be used to compare the results of models that include a static and dynamic trait with models that include only the dynamic trait.

Application of the framework with empirical data is challenging as with all demographic models, as the data requirements are high. Ideally there should be individual-based information on all vital rates and traits considered (age, morphological measures, location, etc). In addition, heritability measures require at least a pedigree, and ideally genetic information. Finally, environmental data on key variables are needed to model and project consequences of environmental change. Depending on the study system and area, environmental data are often readily available, as are climatic models providing scenarios for future changes (Collins *et al*. 2013). Despite these challenges, the valuable insights provided by demographic models into underlying mechanisms make the effort worthwhile (Clutton-Brock & Sheldon 2010). We provided one example here based on a pike population, but even these unique long-term data were not sufficient to estimate all parts of the model. In place of empirical estimates, we made biologically realistic assumptions and tested the effects of varying these (survival effects through *α* and presence or absence of heritability of size at age 1).

Since obtaining such detailed data on individual traits can be highly expensive and difficult, it is important to know the potential consequences of ignoring heterogeneity for parameters of interest. We have demonstrated that besides the population growth rate *λ*, a range of parameters (net reproductive rate, two measures of generation time, demographic variance, environmental variance) can be biased when underlying heterogeneity is ignored, and provided a general method for evaluating these consequences in specific cases. Our theoretical example shows that these biases are generally more severe when all of the structure is ignored, as expected, than if only part of the heterogeneity is ignored. The empirical example for pike demonstrated that the bias can increase with the number of vital rates affected by the static trait, and with the strength of the effect.

Whether or not a biased parameter is a problem, and how much bias can be tolerated, will depend on the specific study goals and on how much other results and conclusions rely on the estimate. Generation time, for instance is an important measure used by, e.g., the IUCN Red List (IUCN, 2014) as one element in categorising the conservation status of a species, including as a scaling factor for other measures. Thus, in the worst case, the assigned conservation status of a species could be wrong if the generation time estimate is biased by ignoring heterogeneity. For cases where conclusions are sensitive to demographic outputs like generation time, extra effort to reveal hidden heterogeneity may therefore be warranted. The direction of the bias in each parameter depends on the underlying heterogeneity as well on as how much of it is ignored. The methods presented here provide a general tool for evaluating consequences of ignoring heterogeneity, also for other parameters than those considered here. The key to this approach is to define an unconditional model with respect to the trait that is ignored.

We have also highlighted different uses of the terms heterogeneity and eco-evolutionary dynamics in the literature, and pointed out the importance of using the same definitions when results of different studies are compared. The conceptual framework introduced here can be used both to evaluate consequences of ignoring heterogeneity, as we have done in this study, and to study consequences of changes in the underlying population, for instance with respect to heterogeneity. In the latter case, it can be challenging to identify the relevant comparison population (i.e. the uniform population to which the heterogeneous population is compared). If the comparison population is defined by having the same average vital rates as in the heterogeneous population, our results regarding consequences of ignoring heterogeneity can also be interpreted as effects of changing heterogeneity. However, this may not always be the relevant comparison. An interesting question regarding changing heterogeneity is whether heterogeneity itself represents an adaptive property of a life history and under which conditions this can evolve (Kendall *et al*. 2011). In this case, the relevant homogeneous comparison population would be that from which heterogeneity evolved. But is this a population where individuals had vital rates equal to the vital rates at birth in the heterogeneous population, or where the vital rates were equal to the average vital rates of the heterogeneous population, or something else? Separating adaptive from non-adaptive heterogeneity will also be a major challenge in such studies. Many different life histories can result in the same optimum fitness (Roff, 1996), that may also display different levels of heterogeneity.

Although it may seem obvious, we must also keep in mind that individual members of a population tend to have much in common, as after all they belong to the same species. Demographic models ignoring heterogeneity often do a good job of describing the population dynamics, at least for the deterministic case in a constant environment (Caswell, 2001). The extent to which individual life histories will differ from the population average may also be restricted by phylogenetic constraints. An elephant, for instance, cannot suddenly display a ‘mouse’ life history where many offspring are produced in one event. In most species, all individuals undergo substantial changes over their lifetime due to ontogenetic development and growth, and they often gain more experience as well. However, we do not yet have a detailed empirical understanding of how individual heterogeneity varies across the tree of life (Metcalf & Pavard 2007), although some differences are apparent. In species of indeterminate growth, in particular, individual heterogeneity due to different growth rates can be substantial (Zuidema *et al*. 2009; de Valpine *et al*. 2014). Recently, Jones *et al*. (2014) published patterns of age-specific mortality and fecundity for a range of species across different taxa, revealing large differences that we are only beginning to understand. Underlying heterogeneity can shape such patterns (Vaupel *et al*. 1979; Caswell, 2014), but may be more or less important for different species. An interesting but challenging task would be to investigate patterns of individual heterogeneity in life history across species, as well as between different populations within species.

Several other applications of the framework are possible. In particular, it could be a powerful tool for investigating eco-evolutionary responses to climatic changes, as illustrated with our pike example. The framework allows key environmental drivers to affect all of the vital rates in different ways, and to interact with the dynamic and static traits. Moreover, consequences of changing environmental variability can be considered as well as changes in the mean. This is particularly relevant in cases where the environmental driver has differing and non-linear effects on the vital rates and population growth rate, which is likely common (García-Carreras & Reuman 2013). Another potential application of the framework is to study the spread of invasive species, for instance to evaluate whether they are able to cross barriers and how fast this may happen. Finally, an interesting topic for future research would be to evaluate impacts of mating system in structured two-sex models (Schindler *et al*. 2013) under varying levels of individual heterogeneity.

## Conclusions

Individual heterogeneity in life histories shape ecological and evolutionary processes, but is often ignored in models applied to study eco-evolutionary dynamics. We propose that separating static and dynamic traits provides a conceptual general framework for individual heterogeneity. The framework is complementary to age–stage-structured models, but specifically includes individual-level properties and requires only one estimate for heritability of each static trait. Demographic models are powerful tools to study the underlying mechanisms of eco-evolutionary dynamics and other processes. The perspective introduced here can provide a useful extension to these approaches, and we hope that future applications may answer some of the many interesting questions that require a detailed knowledge of the mechanisms shaping eco-evolutionary processes.
