# Supplementary material for: Individual heterogeneity in life histories and eco-evolutionary dynamics
Source: Ecol Lett. 2015 Mar 23;18(5):417–32. doi: 10.1111/ele.12421 (PMC4524410; doi:10.1111/ele.12421)
Supplement: Supplementary file 1 [file ele0018-0417-sd1.pdf]

Supporting information -Appendix S1-S4 Individual heterogeneity in life histories and eco-evolutionary dynamics

Yngvild Vindenes* and Øystein Langangen

Centre for Ecological and Evolutionary Synthesis (CEES),
Department of Biosciences, University of Oslo, Oslo, Norway,
*yngvild.vindenes@ibv.uio.no

Overview

Appendix S1: Details of model framework.

Appendix S2: Effects of ignoring heterogeneity.

Appendix S3: Details on pike model and study system.

Appendix S4: Eco-evolutionary dynamics with discrete trait (color example).

Appendix S1: Details of model framework

In this appendix we present in more detail the conceptual model framework including a dynamic trait x and a static trait y, as well as external variables (e.g. temperature or food level) denoted as θ. The framework is based on IPM/matrix models (Caswell, 2001; Easterling *et al.*, 2000; Ellner & Rees, 2007). We present the model using IPM notation for continuous traits, but it can easily be converted for application to discrete traits as well (matrix model, replacing integrals by sums). We begin with a deterministic model, which is later extended to include demographic or environmental stochasticity. Key life history variables and population parameters are calculated using methods from matrix models (Caswell, 2001) and IPMs (Easterling *et al.*, 2000; Ellner & Rees, 2006). A summary of the model parameters and variables is given in table S1.1.

The key features of this model compared to a "standard" IPM/matrix model is the separation between two kinds of traits: static traits are permanent over an individual's lifetime, while dynamic traits change over the lifetime (the most important examples of dynamic traits are age and size). For simplicity we present a model with just one dynamic and one static trait, but the framework can also include cases of more than one of each type. Including a static and dynamic trait requires the definition of a joint distribution for offspring values of the traits, and applying a Dirac delta function (or Kronecker delta in the case of discrete traits) to ensure that the static trait remains constant over the lifetime.

Table S1.1: Summary of parameters and variables used in the model. For discrete traits, integrals are replaced by corresponding sums over the trait values, and the kernels are matrices. A time subscript t is used when necessary.

**Parameter/variable**	**Description**
**Trait distribution**	
x	Dynamic trait (current value or parental value).
x'	Dynamic trait (next value or offspring value).
y	Static trait (current value or parental value).
y'	Static trait (next value y'=y, or offspring value).
Ω_x, Ω_y	Domains of the traits x and y, respectively.
θ	External variable(s)/ environment, e.g. temperature.
$n(x, y)$	Joint population density distribution of the traits (not a probability distribution).
$N = \int_{\Omega_x} \int_{\Omega_y} n(x, y)\mathrm{d}y\mathrm{d}x$	Total population size.
**Vital rates**	
$s(x, y, \theta)$	Survival probability.
$g(x'; x, y, \theta)$	Distribution of x'.
$\delta(y)$	The Dirac delta function.
$b(x, y, \theta)$	Fecundity (offspring number).
$f(x', y'; x, y, \theta)$	Joint trait distribution for offspring traits.
**Kernels**	
$\mathbf{K} = K(x', y'; x, y, \theta)$	Projection kernel (function of all vital rate functions).
$\mathbf{S} = S(x', y'; x, y, \theta)$	Survival kernel.
$\mathbf{B} = B(x', y'; x, y, \theta)$	Reproduction kernel.
$\mathbf{I}$	Identity kernel, same dimension as $\mathbf{K}$ (analogue to identity matrix).
$\mathbf{R} = \mathbf{B}(\mathbf{I} - \mathbf{S})^{-1}$	Net reproduction kernel.
**Demographic outputs**	
λ	Expected long-term population growth rate (average fitness), the dominant eigenvalue of $\mathbf{K}$.
$u(x, y)$	Joint stable structure of x and y, $\int_{\Omega_x} \int_{\Omega_y} u(x, y)\mathrm{d}y\mathrm{d}x = 1$, right eigenfunction associated with λ.
$v(x, y)$	Reproductive values, $\int_{\Omega_x} \int_{\Omega_y} v(x, y)u(x, y)\mathrm{d}y\mathrm{d}x = 1$, left eigenfunction associated with λ.
$V = \int_{\Omega_x} \int_{\Omega_y} v(x, y)n(x, y)\mathrm{d}y\mathrm{d}x$	Total reproductive value.
R_0	Net reproductive rate. Dominant eigenvalue of $\mathbf{R}$.
$G_1 = \ln R_0 / \ln \lambda$	Generation time measured as time for population to grow by a factor R_0.
$G_2 = \frac{\lambda}{\mathbf{v}^T \mathbf{B} \mathbf{u}}$	Generation time measured as mean age of mothers at stable distribution.
σ_e^2	Environmental variance, $\mathrm{Var}(\ln V_{t+1} - \ln V_t \mid V_t)\lambda^2 = \sigma_e^2 + \sigma_d^2 / V_t$.
σ_d^2	Demographic variance.

S1-1. Vital rates and projection function

The population density, describing the expected number of individuals having traits x and y in the population, is given by a joint distribution $n(x, y)$ (not to be confused with a probability distribution), and the total population size is given by $N = \int_{\Omega_y} \int_{\Omega_x} n(x, y) \mathrm{d}x \mathrm{d}y$ (where Ω_x and Ω_y are the sample spaces of x and y, respectively). The change in $n(x, y)$ over time is described by the projection kernel, which is a function of all the vital rates.

For a given value of the environment θ, an individual with current trait values x and y will survive until next year with a probability $s(x, y, \theta)$. Next year it will (if it survived) receive a new dynamic trait value x' according to a distribution $g(x'; x, y, \theta)$. Thus, the static trait y may also influence the transitions of the dynamic trait throughout the entire lifetime of the individual. The individual also produces a number of offspring with expectation $b(x, y, \theta)$, that will enter the population next year. Each offspring will have some trait combination $[x', y']$, which is determined by the distribution $f(x', y'; x, y, \theta)$. This is a joint density distribution for x' and y', recognizing that the traits will often be correlated at birth. In general this distribution may also include heritability (i.e., part of the variation in a trait that is genetically transmitted to offspring; Danchin, 2013). The definition of $f(x', y'; x, y, \theta)$ is flexible and enables modeling of a range of mechanisms for inheritance (for a recent discussion of different mechanisms, see Danchin, 2013), such as additive genetic inheritance (through y), parental effects including epigenetic effects (through x and y and their interaction), or cohort effects (through θ).

The projection kernel is a function of the vital rates describing how the joint distribution of x and y changes from one time step to the next, and is given by

$$K(x',y';x,y,\theta) = S(x',y';x,y,\theta) + B(x',y';x,y,\theta) \tag{S1.1}$$
$$= s(x,y,\theta)g(x';x,y,\theta)\delta(y'-y) + b(x,y,\theta)f(x',y';x,y,\theta),$$

where $\delta(y'-y)$ is the Dirac delta function, described in more detail below. This function ensures that the static trait y does not change over the lifetime of an individual. The projection kernel consists of two main parts, a survival kernel $S(x',y';x,y,\theta)$ and a reproduction kernel $B(x',y';x,y,\theta)$. For a simpler notation, we will denote the kernels using **K** (projection kernel), **S** (survival kernel) **B** (reproduction kernel).

The Dirac delta function is not strictly a function, but can be loosely defined as

$$\delta(x) = \begin{cases} \infty, & x = 0 \\ 0, & x \neq 0, \end{cases}$$
$$\int_{\{A\}} \delta(x)\mathrm{d}x = 1, \;\; 0 \in A \; ,$$

with the important property that

$$\int_{\{A\}} f(x)\delta(x-x_0)\mathrm{d}x = f(x_0) \text{ if } x_0 \in A \; .$$

A reduced model treating the static trait as a parameter

It can sometimes be useful to consider a reduced version of the model, where the static trait y is treated as a parameter influencing the life history rather than a state variable. This corresponds to a model assuming the entire population has

the same value of y which is perfectly inherited, as for instance in ESS (evolutionary stable strategy) analyses where the trait is varied to find a strategy (i.e. life history) that cannot be "invaded" by other strategies. The projection kernel of the reduced model is given by

$$\begin{aligned} K(x'; x, y, \theta) &= S(x'; x, y, \theta) + B(x'; x, y, \theta) \\ &= s(x, y, \theta) g(x'; x, y, \theta) + b(x, y, \theta) f_x(x'; x, y, \theta). \end{aligned}$$

The assumption that all individuals have the same value of y may be more reasonable for studies comparing macro-evolutionary (long-term) patterns, such as ESS analyses, than for micro-evolutionary (i.e. short term, eco-evolutionary) processes where variation in y can be an important factor. However, due to the reduced dimensionality such a model is computationally easier to explore than the full model including variation in y, and may therefore be useful at least for initial explorations.

S1-2. Demographic outputs calculated from the deterministic model

Long-term growth rate, stable trait structure, and reproductive value

The projection function $\mathbf{K}$ can be analyzed using standard matrix methods (after discretization; Caswell, 2001; Ellner & Rees, 2006; Rees *et al.*, 2014) to obtain the long-term population growth rate λ (the dominant eigenvalue of $\mathbf{K}$), the stable structure $u(x, y)$ (right eigenfunction) and reproductive value function $v(x, y)$ (left eigenfunction), scaled so that $\int_{\Omega_y} \int_{\Omega_x} u(x, y) \mathrm{d}x \mathrm{d}y = 1$ and $\int_{\Omega_y} \int_{\Omega_x} v(x, y) u(x, y) \mathrm{d}x \mathrm{d}y = 1$. We generally assume that all assumptions

required for a unique λ to exist are met (Caswell, 2001; Ellner & Rees, 2006).

Net reproductive rate

The net reproductive rate R_0 is the expected lifetime reproduction of an individual, and a measure of the generation-to-generation population growth rate (Caswell, 2001). When offspring are born with different trait values (of x and/or y), the expected lifetime reproduction is conditional on the traits at birth. The generation to generation population growth is described by the kernel $\mathbf{R} = \mathbf{B}(\mathbf{I} - \mathbf{S})^{-1}$ (Ellner & Rees, 2006), where $\mathbf{B}$ is the reproduction kernel as defined above, $\mathbf{S}$ is the survival/transition kernel, and $\mathbf{I}$ is the identity kernel (analogue to the identity matrix). Additional requirements need to be fulfilled for the existence of a unique dominant eigenvalue R_0 for this kernel, which defines the net reproductive rate (Section 5.3.4 in Caswell, 2001).

Generation time

Generation time is an important property used to characterize life histories. Several processes also scale with generation time, both evolutionary (e.g. the fixation probability of a new allele) and ecological (e.g. extinction).

Generation time is intuitively understood as the time between generations, but for structured populations with overlapping generations the definition is not straightforward. Different measures have been used, and they usually produce slightly different values, depending on the life history (Caswell, 2001; Bienvenu *et al.*, 2013). We consider two of these measures. The first is the time it takes for the population to increase by a factor R_0, $G_1 = \ln R_0 / \ln \lambda$, while the second is the mean age of mothers in a population at the stable structure. In the heterogeneous population this is given by (Bienvenu *et al.*, 2013) $G_2 = \frac{\lambda}{\mathbf{v}^T \mathbf{B} \mathbf{u}}$.

S1-3. Including stochasticity

Demographic and environmental stochasticity in unstructured populations

Natural populations will show random fluctuations due to stochasticity in the environment as well as inherent randomness in the individual events of survival, reproduction and trait transitions (in addition to observation errors, which we ignore here). Environmental stochasticity represents fluctuations in the annual growth rate arising from environmental effects that are common for all individuals, while demographic stochasticity arises from random variation among individuals in the events of survival, reproduction, and state transitions (May, 1973b; Lande *et al.*, 2003).

For an unstructured density-independent population process (assuming no autocorrelation in the environment), the population size N follows a Markovian process, where the variance in population growth can be decomposed as (Engen *et al.*, 1998)

$$\mathrm{Var}(\Delta N | N_t) = \sigma_e^2 N_t^2 + \sigma_d^2 N_t,$$

where $\Delta N = N_{t+1} - N_t$. The constants σ_e^2 and σ_d^2 are denoted the environmental variance and demographic variance, respectively, assuming no demographic covariance between individuals. Using a similar approach of Engen *et al.* (2007) by letting $N_{t+1} = \lambda N_t + \varepsilon$, where $\mathrm{E}[\varepsilon] = 0$ and $\mathrm{Var}(\varepsilon) = \mathrm{Var}(\Delta N | N_t)$, we have that

$$
\begin{aligned}
N_{t+1} &= \lambda N_t \left(1 + \frac{\varepsilon}{\lambda N_t}\right) \\
\ln N_{t+1} &= \ln \lambda + \ln N_t + \ln \left(1 + \frac{\varepsilon}{\lambda N_t}\right) \\
&= \ln \lambda + \ln N_t + \sum_{k=1}^{\infty} (-1)^k \frac{\left(\frac{\varepsilon}{\lambda N_t}\right)^k}{k},
\end{aligned}
$$

where the last sum is a Taylor expansion of the term $\ln(1 + \varepsilon/(\lambda N_t))$. Including terms up to the second order, we get the expression

$$
\ln N_{t+1} - \ln N_t \approx \ln \lambda - \frac{\varepsilon}{\lambda N_t} + \frac{1}{2} \left(\frac{\varepsilon}{\lambda N_t}\right)^2 .
$$

The long-term (stochastic) growth rate is the expectation of $\ln N_{t+1} - \ln N_t$ (conditional on the current population size N_t), and is given by

$$
\begin{aligned}
r_s = \mathrm{E}[\ln N_{t+1} - \ln N_t | N_t] &\approx \ln \lambda + \frac{\mathrm{E}[\varepsilon^2]}{2\lambda^2 N_t^2} \\
&= \ln \lambda - \frac{\mathrm{Var}(\varepsilon)}{2\lambda^2 N_t^2} \\
&= \ln \lambda - \frac{1}{2\lambda^2} \sigma_e^2 - \frac{1}{2\lambda^2 N_t} \sigma_d^2 .
\end{aligned}
$$

Thus, unless the demographic variance is zero the stochastic growth rate is a function of N_t and increases with increasing population size (Lande *et al.*, 2003). Assuming the expectation $E[\varepsilon^4]$ is so small that it can be ignored, the variance of the population growth increment on log scale is given by

$$\text{Var}(\ln N_{t+1} - \ln N_t | N_t) \approx \frac{1}{\lambda^2 N_t^2}\text{Var}(\varepsilon) = \frac{1}{\lambda^2}\sigma_e^2 + \frac{1}{\lambda^2 N_t}\sigma_d^2.$$

Stochasticity in structured populations

Several studies contributed to develop the theory of stochastic dynamics in structured populations, both considering demographic stochasticity (Bartlett, 1960) and environmental stochasticity (Lewontin & Cohen, 1969; May, 1973a; Turelli, 1977; Tuljapurkar, 1990). Later, Engen *et al.* (2005) combined the two types of stochasticity in an age-structured model, and demonstrated that the three parameters λ, σ_d^2 and σ_e^2 could be used to define an accurate diffusion approximation (a continuous time approximation of the discrete time population process) of the population dynamics, extending the result of Lande & Orzack (1988) to include demographic stochasticity. This demonstrated that these three parameters alone contain the necessary information to describe the population dynamics even with complex life histories. A number of results are also available from diffusion models that enables prediction of extinction risk and characterization of time to extinction (Karlin & Taylor, 1981). By a first order approximation they also showed that the stochastic growth rate takes the same form as in the unstructured case, extending the small-noise approximation of Tuljapurkar (1982) to include demographic stochasticity.

Describing the stochastic dynamics of structured populations is complicated by the fact that the population size will be autocorrelated due to transient fluctuations in the trait distribution, so that N is no longer Markovian as in the unstructured case. Engen *et al.* (2007) showed how the dynamics could be described by considering the total reproductive value V, calculated using the reproductive values of the mean environment. This quantity will follow the

process of N, and the dynamics are approximately Markovian, just as in the unstructured case. Thus, the reproductive values work as a filter removing the transient fluctuations in the trait distribution, and the environmental and demographic variance can be calculated from this process. The above equations for the unstructured case are basically the same in the structured case, replacing N by V (Engen *et al.*, 2007).

Stochasticity in IPMs

Both environmental stochasticity (Ellner & Rees, 2007; Rees & Ellner, 2009; Vindenes *et al.*, 2011) and demographic stochasticity (Vindenes *et al.*, 2011, 2012) have been included in IPMs. Vindenes *et al.* (2011) demonstrated how their effects can be separated when both types of stochasticity are included at the same time, and extended the results of Engen *et al.* (2005) to general population structure. As in matrix models, stochasticity in IPMs are included through the individual vital rates.

The effects of environmental stochasticity can be estimated through the regression models (e.g. mixed models) for the vital rates (Ellner & Rees, 2007), via specific environmental drivers (explained variation and covariation), via random year effects (unexplained variation and covariation), or both (e.g. temperature and random year effects; Vindenes *et al.*, 2014). An important question to consider is at which scale the vital rates are modeled (e.g., the log scale, logit scale, square root squale, or absolute scale), as the environmental random effects are often assumed to be normally distributed on that scale. This normality assumption can be checked, for instance with diagnostic plots from the regressions or specific tests. If these show that the normality assumption is not met, another scale transformation may be needed.

Demographic stochasticity arises from inherent stochasticity in the processes

of survival, transition and offspring production. For some of these processes the variance is always given by the mean. For instance survival is always a binomial process and the demographic variance in survival is entirely described by the survival probability. The variance in offspring number can be a function of the mean, but the distribution of offspring number will vary depending on species and population. If individual data on reproduction are lacking, the best we can do is to make some assumption on the nature of the distribution of offspring number, based on knowledge from other populations or from general knowledge of the life history of the species. Demographic variance in trait transitions largely depend on the type of trait. In matrix models trait transitions always follow a multinomial process, but calculating the probability of each possible event is not always straightforward. In IPMs, transitions in size (growth) are often assumed to follow a normal distribution, which may not always be biologically reasonable (although such a model may still provide an adequate description of the average dynamics). A normal distribution is not ideal as in theory it could produce negative body sizes, as well as impose shrinking when this is not necessarily biologically reasonable. Thus, defining a reasonable distribution for variance in dynamic transitions is not straightforward, and should be done with some care.

Demographic and environmental variance in IPMs

The demographic and environmental variance partition the variance in population growth, but can also be defined at the individual level (Engen *et al.*, 2009). The demographic variance σ_d^2 can be defined as the expected variance in an individual contribution to next year's total reproductive value (expectation with respect to environment, and variance with respect to individual contributions), while the environmental variance σ_e^2 can be defined as the variance of the expected contribution (variance with respect to environment, and

expectation with respect to individual contributions Engen *et al.*, 2009; Vindenes *et al.*, 2012). Ignoring environmental stochasticity (to simplify notation), the demographic variance of our model is given by (Vindenes *et al.*, 2011)

$$\sigma_d^2 \approx \int_{\Omega_y} \int_{\Omega_x} u(x,y)[\mu_{VS}^2(x,y)\sigma_S^2(x,y) + \mu_{VB}^2(x,y)\sigma_B^2(x,y) \\ + 2\mu_{VS}(x,y)\mu_{VB}(x,y)\sigma_{BS}^2(x,y) + \sigma_{VS}^2(x,y)s(x,y) + \sigma_{VB}^2 b(x,y)]\mathrm{d}x\mathrm{d}y.$$

where $\sigma_S^2(x,y) = s(x,y)(1-s(x,y))$, $\sigma_B^2(x,y)$ is the variance in offspring number, $\sigma_{BS}^2(x,y)$ is the covariance between survival and reproduction, $\mu_{VS}(x,y)$ and $\sigma_{VS}^2(x,y)$ are the expectation and variance, respectively, of next year's reproductive value for an individual with states x and y, and $\mu_{VB}(x,y)$ and $\sigma_{VB}^2(x,y)$ are the corresponding parameters for an offspring. These are given by

$$\begin{aligned}
\mu_{VS}(x,y) &= \int_{\Omega_x} v(x',y)g(x';x,y)\mathrm{d}x', \\
\sigma_{VS}^2(x,y) &= \int_{\Omega_x} v^2(x',y)g(x';x,y)\mathrm{d}x' - \mu_{VS}^2(x,y), \\
\mu_{VB}(x,y) &= \int_{\Omega_x} \int_{\Omega_y} v(x',y')f(x',y';x,y)\mathrm{d}x'\mathrm{d}y', \\
\sigma_{VB}^2(x,y) &= \int_{\Omega_x} \int_{\Omega_y} v^2(x',y')f(x',y';x,y)\mathrm{d}x'\mathrm{d}y' - \mu_{VB}^2(x,y).
\end{aligned}$$

Thus, the reproductive value plays an important role in the definition of the demographic variance. When demographic stochasticity is ignored (large populations), the environmental variance as a function of the individual vital rates is given by (Vindenes *et al.*, 2011)

$$\sigma_e^2 \approx \int_{\Omega_y}\int_{\Omega_x}\int_{\Omega_y}\int_{\Omega_x} u(x_1,y_1)u(x_2,y_2)\mathrm{Cov}\left(w(x_1,y_1),w(x_2,y_2)\right)\mathrm{d}x_1\mathrm{d}y_1\mathrm{d}x_2\mathrm{d}y_2,$$

where $w(x_1, y_1)$ is the contribution of an individual with trait value $[x_1, y_1]$ to next year's total reproductive value, while $w(x_2, y_2)$ is the contribution from an individual of traits $[x_2, y_2]$. Estimating this covariance across all combinations of trait values is generally difficult. Instead, we estimate σ_e^2 from simulation of the process of V, using (Engen *et al.*, 2007; Vindenes *et al.*, 2011)

$$r_s = E[\ln V_{t+1} - \ln V_t|V_t] = \lim_{t\to\infty}\frac{1}{t}\sum_{i=1}^{t}(\ln V_i - \ln V_{i-1}),$$
$$\sigma_e^2 = \lambda^2\mathrm{Var}(\ln V_{t+1}|V_t) = \lambda^2\lim_{t\to\infty}\frac{1}{t-1}\sum_{i=1}^{t}(\ln V_i - \ln V_{i-1} - r_s)^2.$$

Extinction risk from diffusion approximation

Based on the diffusion approximation one can also calculate the risk of ultimate extinction U starting from a population size N_0 (Karlin & Taylor, 1981). For $r_s < 1$ this probability is 1, but for $r_s > 1$ the probability of extinction at $N = 1$ is given by (Engen *et al.*, 2005)

$$P(U|N_0) = \left(\frac{\sigma_d^2 + \sigma_e^2}{\sigma_d^2 + \sigma_e^2 N_0}\right)^{2r_s/\sigma_e^2},$$

starting from a population of size N_0. When both demographic and environmental stochasticity is included there is no simple expression for the distribution of time to extinction, T_E. When demographic stochasticity is ignored, the time to extinction follows an inverse Gaussian distribution (Karlin &

Taylor, 1981; Engen *et al.*, 2005). If environmental stochasticity is ignored (only demographic stochasticity) the cumulative distribution of time to extinction at $N = 0$, conditional on starting at population size N_0, is given by (Cox & Miller, 1965; Engen *et al.*, 2005)

$$P(T_E < t|N_0) = \exp\left(-\frac{2N_0 \ln \lambda}{\sigma_d^2(1-\lambda^{-t})}\right).$$

S1-4. Notes on numerical implementation

All calculations were done with the software R (R Development Core Team, 2013). We have provided R code for all our examples as supplementary material, where additional plots of various parts of the model are also provided. This code also demonstrates how the IPM can be discretized for numerical calculations. Detailed procedures for general numerical implementation of IPMs are also described elsewhere (for instance Merow *et al.*, 2014; Rees *et al.*, 2014).

The discretization of an IPM is generally done after the vital rates have been estimated using regression, turning the model into a large matrix model before calculation of model parameters (Ellner & Rees, 2006). The dimension of the model (the number of "mesh points") determines the accuracy of the results, but computation efficiency can be reduced if the dimensions of the matrix is very large. This can lead to long computing times in particular if multiple matrix operations are involved, for instance in stochastic models. For initial exploration of the models we recommend using a lower dimension, and then for the final calculations to use a higher dimension. The dimension should be high enough that results are not altered (to a desired degree of accuracy) if it is increased further. To discretize the model we divide the trait space of the dynamic trait x into m stages (each of length Δx) and the trait space of the static trait y into n

stages (each of length Δy). The vital rate functions for survival and fecundity are discretized into vectors of length mn (stacking the trait vectors), whereas the transition functions are discretized into matrices of dimension $mn \times mn$. The resulting projection matrix is also a $mn \times mn$ projection matrix.

Appendix S2: Effects of ignoring heterogeneity

In this appendix we derive the results on consequences of ignoring heterogeneity presented in the main text. Here we assume that the underlying heterogeneous population (the "true" process) is defined by the model including a static trait y and a dynamic trait x, defined above. We then consider to the general cases where either all of the heterogeneity (i.e. both traits x and y) is ignored, or just part of the heterogeneity is ignored (i.e. ignoring the static trait y but still including x).

For each of the two cases we need to derive the corresponding model applying to the same underlying heterogeneous population. Such a model will be an unconditional model (i.e. unconditional with respect to y, or to x and y) and its parameters can therefore be calculated as functions of the parameters of the underlying model, applying formulas for unconditional mean, variance and covariance. The case where all structure is ignored corresponds, naturally, to an unstructured model, which is a simpler case than that of ignoring only part of the heterogeneity. In the latter case, the model will still be structured in x and new transition distributions for this trait must also be derived. Vindenes *et al.* (2008) applied the approach to study consequences of heterogeneity for demographic variance, but did not consider the case where only part of heterogeneity is ignored. Here we extend the approach to consider such cases, and also apply the approach to a range of parameters in addition to the demographic variance.

In this appendix we first derive the two comparison models ignoring all or part of the heterogeneity. In the following sections we apply these models to evaluate the consequences of ignoring heterogeneity for each of the demographic outputs mentioned in Appendix S1 (the long-term growth rate λ, the net reproductive rate R_0, generation time G_1 and G_2, demographic variance σ_d^2 and environmental variance σ_e^2, and extinction risk).

To help distinguish parameters from the heterogeneous and homogeneous models, we use a notation where parameters representing the model ignoring y are marked with an asterisk * (when necessary), while parameters for the model ignoring both x and y are marked with a double asterisk **. For simplicity, we do not include the environmental variable θ in the equations here, but every vital rate can in principle depend on this variable as well.

S2-1. Comparison model ignoring all heterogeneity

From the heterogeneous model (model structured according to x and y) we have the joint stable trait distribution $u(x, y)$ (scaled so that $\int_{\Omega_y}\int_{\Omega_x} u(x, y)\mathrm{d}x\mathrm{d}x = 1$). This function can be considered as the joint probability distribution for sampling an individual with trait combination x and y (from a large population at stable distribution). Denoting the events of survival and reproduction (offspring number) as S and B, respectively, the estimates for survival probability and fecundity in this homogenous model are (Vindenes *et al.*, 2008)

$$s = \mathrm{E}[S|x, y] = \int_{\Omega_y}\int_{\Omega_x} u(x, y)s(x, y)\mathrm{d}x\mathrm{d}y,$$
$$b = \mathrm{E}[B|x, y] = \int_{\Omega_y}\int_{\Omega_x} u(x, y)b(x, y)\mathrm{d}x\mathrm{d}y.$$

These values are weighted averages, weighted by the stable structure. For the deterministic case, these are all the parameters needed to define the model, and the population growth rate is given by $\lambda = s + b$ (which is exactly the same as in the heterogeneous model, as shown later).

To calculate the demographic variance, three more parameters need to be defined describing the unconditional variance and covariance of survival and fecundity. These are given by (Vindenes *et al.*, 2008)

$$\begin{aligned}
\sigma_S^2 &= \mathrm{E}[\mathrm{Var}(S|x,y)] + \mathrm{Var}(\mathrm{E}[S|x,y]) \\
&= \int_{\Omega_y}\int_{\Omega_x} u(x,y)s(x,y)(1-s(x,y))\mathrm{d}x\mathrm{d}y + \int_{\Omega_y}\int_{\Omega_x} u(x,y)(s(x,y)-s)^2\mathrm{d}x\mathrm{d}y \\
&= s(1-s),
\end{aligned}$$

$$\begin{aligned}
\sigma_B^2 &= \mathrm{E}[\mathrm{Var}(B|x,y)] + \mathrm{Var}(\mathrm{E}[B|x,y]) \\
&= \int_{\Omega_y}\int_{\Omega_x} u(x,y)[\sigma_B^2(x,y) + (b(x,y)-b)^2]\mathrm{d}x\mathrm{d}y,
\end{aligned}$$

and

$$\begin{aligned}
\sigma_{BS}^2 &= \mathrm{E}[\mathrm{Cov}(B,S|x,y)] + \mathrm{Cov}(\mathrm{E}[S|x,y],\mathrm{E}[B|x,y]) \\
&= \int_{\Omega_y}\int_{\Omega_x} u(x,y)[\sigma_{BS}^2(x,y) + (b(x,y)-b)(s(x,y)-s)]\mathrm{d}x\mathrm{d}y.
\end{aligned}$$

Thus, even if the covariance from the heterogeneous model $\sigma_{BS}^2(x,y)$ is zero, the covariance can be non-zero in the homogeneous model ignoring heterogeneity.

S2-2. Comparison model ignoring part of the heterogeneity

Many models account for some variation among individuals, for instance due to age or size, but other sources of heterogeneity may still be ignored. The derivation of a model ignoring the variation in trait y but keeping the trait x follows the same approach as above, but for this model we also need to derive the transition functions for the trait x that is still included.

Using the joint stable distribution $u(x, y)$ from the heterogeneous model together with standard formulas for manipulation of conditional probabilities and expectations (in particular Bayes' theorem, the multiplication rule, and the law of total probability), obtaining expressions for the vital rates unconditional on y is relatively straightforward. As before, let the events of survival and reproduction (offspring number) be denoted as S and B, respectively. The unconditional survival probability and fecundity as a function of x are given by

$$\begin{aligned} s(x) = E(S|x) = \frac{\int_{\Omega_y} E(S|x,y)P(x,y)\mathrm{d}y}{P(x)} &= \frac{\int_{\Omega_y} s(x,y)u(x,y)\mathrm{d}y}{\int_{\Omega_y} u(x,y)\mathrm{d}y}, \\ b(x) = E(B|x) = \frac{\int_{\Omega_y} E(B|x,y)P(x,y)\mathrm{d}y}{P(x)} &= \frac{\int_{\Omega_y} b(x,y)u(x,y)\mathrm{d}y}{\int_{\Omega_y} u(x,y)\mathrm{d}y}, \end{aligned}$$

where $P(x, y)$ and $P(x)$ are the probabilities of selecting an individual of trait values (x, y) and x, respectivily. The former probability is given by $u(x, y)$ while the latter is given by the marginal distribution of x, $u(x) = \int_{\Omega_y} u(x, y)\mathrm{d}y$. The distributions for transition in x are slightly more complex to calculate from the heterogeneous model, because transitions are conditional on survival. Thus, the distribution $g(x'; x)$ in this homogeneous model will depend not only on the distribution $g(x'; x, y)$ but also on the survival function $s(x, y)$. We derive this transition function by considering the survival kernel, which specifies the

expected number of individuals with trait values x, y that survive and obtain the trait values x', y' (where $y' = y$) in the next time step. The survival kernel of the model ignoring y is obtained by integrating the survival kernel over y' (to obtain the marginal distribution of x'), and applying the law of total expectation, i.e.

$$
\begin{aligned}
g(x';x)s(x) &= \frac{\int_{\Omega_y}\int_{\Omega_y}\delta(y'-y)g(x';x,y)s(x,y)u(x,y)\mathrm{d}y\mathrm{d}y'}{\int_{\Omega_y}u(x,y)\mathrm{d}y},\\
g(x';x) &= \frac{\int_{\Omega_y}g(x';x,y)s(x,y)u(x,y)\mathrm{d}y\int_{\Omega_y}u(x,y)\mathrm{d}y}{\int_{\Omega_y}s(x,y)u(x,y)\mathrm{d}y\int_{\Omega_y}u(x,y)\mathrm{d}y}\\
&= \frac{\int_{\Omega_y}g(x';x,y)s(x,y)u(x,y)\mathrm{d}y}{\int_{\Omega_y}s(x,y)u(x,y)\mathrm{d}y}.
\end{aligned}
$$

This expression assumes $s(x, y) > 0$, otherwise $g(x'; x) = 0$ for that value of x. As in the heterogeneous model, the transition function $g(x'; x)$ satisfies the condition $\int_{\Omega_x} g(x'; x)\mathrm{d}x' = 1$.

To calculate the offspring distribution of x' when y is ignored we follow the same general procedure applied on the reproduction kernel, which specifies the expected number of offspring produced by an individual of trait values x, y that obtain the trait values x', y'. To obtain the reproduction kernel for the model ignoring y we integrate over y' in the reproduction kernel (to obtain the marginal distribution of x') and apply the law of total expectation, i.e.

$$\begin{aligned}
f(x';x)b(x) &= \frac{\int_{\Omega_y}\int_{\Omega_y} f(x',y';x,y)b(x,y)u(x,y)\mathrm{d}y\mathrm{d}y'}{\int_{\Omega_y} u(x,y)\mathrm{d}y}, \\
f(x';x) &= \frac{\int_{\Omega_y}\int_{\Omega_y} f(x';x,y)b(x,y)u(x,y)\mathrm{d}y\mathrm{d}y' \int_{\Omega_y} u(x,y)\mathrm{d}y}{\int_{\Omega_y} b(x,y)u(x,y)\mathrm{d}y \int_{\Omega_y} u(x,y)\mathrm{d}y} \\
&= \frac{\int_{\Omega_y}\int_{\Omega_y} f(x';x,y)b(x,y)u(x,y)\mathrm{d}y\mathrm{d}y'}{\int_{\Omega_y} b(x,y)u(x,y)\mathrm{d}y}.
\end{aligned}$$

This expression assumes $b(x,y) > 0$, otherwise $f(x';x) = 0$ for that value of x. The offspring trait distribution also satisfies the condition $\int_{\Omega_x} f(x';x)\mathrm{d}x' = 1$. The projection kernel of this model ignoring y is given by

$$K(x';x) = s(x)g(x';x) + b(x)f(x';x).$$

The long-term growth rate λ of this model is also the same as in the heterogeneous model, and the stable distribution $u(x)$ corresponds to the marginal distribution of x in the heterogeneous model, $u(x) = \int_{\Omega_y} u(x,y)\mathrm{d}y$. The reproductive values $v(x)$ of this model, however, do not generally correspond to the average reproductive values (average with respect to y) in the heterogeneous model, and should be calculated from the projection kernel.

Using the same approach as in the previous subsection, the demographic variance components for survival, fecundity, and the covariance between survival and offspring are given by

$$
\begin{aligned}
\sigma_S^2(x) &= \frac{\int_{\Omega_x} u(x,y)\,[\sigma_S^2(x,y) + [s(x,y) - s(x)]^2]\,\mathrm{d}y}{u(x)} = s(x)(1 - s(x)), \\
\sigma_B^2(x) &= \frac{\int_{\Omega_y} u(x,y)\,[\sigma_B^2(x,y) + [b(x,y) - b(x)]^2]\,\mathrm{d}y}{u(x)}, \\
\sigma_{BS}^2(x) &= \frac{\int_{\Omega_y} u(x,y)\,[\sigma_{BS}^2(x,y) + [s(x,y) - s(x)][b(x,y) - b(x)]]\,\mathrm{d}y}{u(x)}.
\end{aligned}
$$

For this model calculation of demographic variance also requires that we consider demographic stochasticity arising from transitions in x and in the process of offspring allocation of x. Given the distributions $g(x'; x)$ and $f(x'; x)$, and the reproductive values $v(x)$ calculated from the projection kernel above, we have

$$
\begin{aligned}
\mu_{VS}(x) &= \int_{\Omega_x} v(x')g(x'; x)\mathrm{d}x', \\
\sigma_{VS}^2(x) &= \int_{\Omega_x} v^2(x')g(x'; x)\mathrm{d}x' - \mu_{VS}^2(x), \\
\mu_{VB}(x) &= \int_{\Omega_x} v(x')f(x'; x)\mathrm{d}x', \\
\sigma_{VB}^2(x) &= \int_{\Omega_x} v^2(x')f(x'; x)\mathrm{d}x' - \mu_{VB}^2(x).
\end{aligned}
$$

S2-3. Consequences of ignoring heterogeneity

In this section we use the comparison models to evaluate the consequences of ignoring heterogeneity for different model outputs, by comparison with the full heterogeneous model.

Consequences for the growth rate λ

Whether or not the underlying heterogeneity is recognized does not affect the value of the expected growth rate λ. This follows from the well known result in matrix models that when the stable distribution is reached, all stages will grow with the same rate λ (Leslie, 1945; Caswell, 2001). Thus, we should see the same growth rate λ no matter how the stages are defined.

Here we demonstrate this result using the model ignoring both x and y, although it holds also for the case where only y is ignored. Here, λ is given by

$$\lambda = s + b = \int_{\Omega_y} \int_{\Omega_x} u(x, y)[s(x, y) + b(x, y)]\mathrm{d}x\mathrm{d}y.$$

To prove that this is the same as in the heterogeneous model, consider the equation for the right eigenfunction (Caswell, 2001), $\int_{\Omega_y} \int_{\Omega_x} K(x', y', x, y)u(x, y)\mathrm{d}x'\mathrm{d}y' = \lambda u(x, y)$. From this, and using the properties $\int_{\Omega_x} \int_{\Omega_y} g(x'; x, y)\delta(y' - y)\mathrm{d}x'\mathrm{d}y' = \int_{\Omega_y} \int_{\Omega_x} f(x', y'; x, y)\mathrm{d}x'\mathrm{d}y' = \int_{\Omega_y} \int_{\Omega_x} u(x, y)\mathrm{d}x\mathrm{d}y = 1$, we have

$$\begin{aligned}\int_{\Omega_y} \int_{\Omega_x} \lambda u(x, y)\mathrm{d}x\mathrm{d}y = \int_{\Omega_y} \int_{\Omega_x} \int_{\Omega_y} \int_{\Omega_x} & u(x, y)[s(x, y)g(x'; x, y)\delta(y - y') \\ & + b(x, y)f(x', y'; x, y)]\mathrm{d}x'\mathrm{d}y'\mathrm{d}x\mathrm{d}y,\end{aligned}$$

$$
\begin{aligned}
\lambda &= \int_{\Omega_y}\int_{\Omega_x} u(x,y)[s(x,y)+b(x,y)]\left[\int_{\Omega_y}\int_{\Omega_x}[g(x';x,y)\delta(y-y')+f(x',y';x,y)]\mathrm{d}x'\mathrm{d}y'\right]\mathrm{d}x\mathrm{d}y \\
&= \int_{\Omega_y}\int_{\Omega_x} u(x,y)[s(x,y)+b(x,y)]\mathrm{d}x\mathrm{d}y \\
&= s+b=\lambda.
\end{aligned}
$$

Thus, ignoring heterogeneity does not influence our estimate of long-term growth rate λ. We emphasize that this result applies to cases of hidden heterogeneity, i.e. where the underlying population (the "true process") is the same, and only the model we use to describe it is changing. If the underlying population is not the same, i.e. when different populations are compared, any model parameter can be affected, including λ (Kendall *et al.*, 2011).

Consequences for the net reproductive rate R_0

For the model ignoring all heterogeneity, we have $R_0^{**} = b/(1-s)$. We could also find this using the standard formula for age-structured models (e.g. Caswell, 2001). Letting a denote age class, $R_0^{**} = \sum_{a=1}^{\infty} s^{(a-1)}b = b/(1-s)$ (where the last equality is applying a standard formula for geometric series).

For the comparison model ignoring only y, R_0^{*} is found in the same way as in the heterogeneous model, using the formula $\mathbf{R}^* = \mathbf{B}^*(\mathbf{I}-\mathbf{S}^*)^{-1}$, where the kernels $\mathbf{S}^*$ and $\mathbf{B}^*$ are now defined from the vital rates of this model.

From this we see that in general the net reproductive rate will be affected by ignoring heterogeneity, but the amount and direction of the bias will depend on the details of the underlying model. Some examples are provided in the main text.

Consequences for generation time

Since λ is not affected by ignoring heterogeneity, the effect on the first generation time estimate $G_1 = \ln R_0 / \ln \lambda$ is determined entirely by the effect on R_0. Thus, for the comparison models we have $G_1^{**} = \ln R_0^{**} / \ln \lambda$ and $G_1^{*} = \ln R_0^{*} / \ln \lambda$.

For the other measure of generation time, mean age of mothers at stable distribution, ignoring all heterogeneity gives the estimate $G_2^{**} = \lambda / b$. Again, using the age-structured measure we get $G_2^{**} = \sum_{a=1}^{\infty} b \lambda^{-a} s^{(a-1)}$ (Caswell, 2001), and from the formula of the sum of a geometric series this is also equal to λ / b.

For the model ignoring only y we have $G_2^{*} = \frac{\lambda}{\mathbf{v}^{*\mathbf{T}} \mathbf{B}^{*} \mathbf{u}^{*}}$. Thus, here the effects of heterogeneity on generation time are mediated through the effect on reproductive value $v(x)$ and the fecundity/offspring transition kernel $B(x', x)$. As reproductive values are determined by all vital rates, including survival and transitions in x, consequences of ignoring heterogeneity in these rates for generation time will be mediated through the reproductive value.

Consequences for the demographic variance

Effects of heterogeneity on demographic variance was studied by Vindenes *et al.* (2008) using matrix models, but not for the case ignoring just part of the heterogeneity. For the homogeneous model ignoring both x and y the demographic variance is given by

$$\sigma_d^{2**} = \sigma_S^2 + \sigma_B^2 + 2\sigma_{BS}^2.$$

while the demographic variance of the model ignoring y is given by

$$\sigma_d^{2*} = \int_{\Omega_x} u(x) \left[s(x)\sigma_{VS}^2(x) + b(x)\sigma_{VB}^2(x) + \sigma_S^2(x)\mu_{VS}^2(x) \right.$$
$$\left. + \sigma_B^2(x)\mu_{VB}^2(x) + 2\sigma_{BS}^2(x)\mu_{VS}(x)\mu_{VB}(x) \right] \mathrm{d}x.$$

Vindenes *et al.* (2008) showed that the effects of ignoring heterogeneity on demographic variance could be both positive and negative, depending on the details of the underlying model and the combination of heterogeneity in survival, fecundity, and transitions.

Consequences for the environmental variance

Engen *et al.* (2007) emphasized that ignoring age structure could lead to serious bias in the estimation of environmental variance because fluctuations due to transient dynamics of the age structure are not accounted for. The same is true with any type of heterogeneity, and we can measure the effect through the total reproductive value (at least as an approximation).

For the comparison model ignoring all heterogeneity, we would calculate the environmental variance based on just the time series of population sizes N, (wrongly) assuming these followed a Markovian process. For the other model ignoring variation in y but not in x, we would calculate the environmental variance based on the time series of $V^* = \int_x v(x)n(x)\mathrm{d}x$, the measure of total reproductive value for this model (for the model ignoring all heterogeneity the total reproductive value is simply $V^{**} = N$. Thus, the severity of ignoring heterogeneity in y on environmental variance depends on how much the reproductive values are affected.

The direction of the bias in environmental stochasticity introduced by ignoring heterogeneity depends on how the stochasticity enters the model, in

particular on which vital rates are affected. In general, with stochasticity in fecundity the environmental variance is likely to be overestimated when heterogeneity is ignored, and vice versa for stochastic survival (Engen *et al.*, 2007). Some examples are provided in the supplementary R code for the theoretical color example. The bias can be large, depending on the underlying heterogeneity.

Consequences for extinction risk

Ignoring heterogeneity affects the risk of ultimate extinction conditional on current population size $P(U|N_0)$ through the demographic and environmental variance. Thus, to the extent that ignoring heterogeneity affects σ_d^2 and σ_e^2, it will also affect estimates of extinction risk through these parameters. The estimated distribution of time to extinction will also generally be affected. Again, the direction and magnitude of the bias will depend on the underlying model. In the supplementary R code for the theoretical color example we provide an example of the different extinction risks calculated when all or part of the heterogeneity is ignored. One example is shown here in figure 1, with environmental stochasticity in survival.

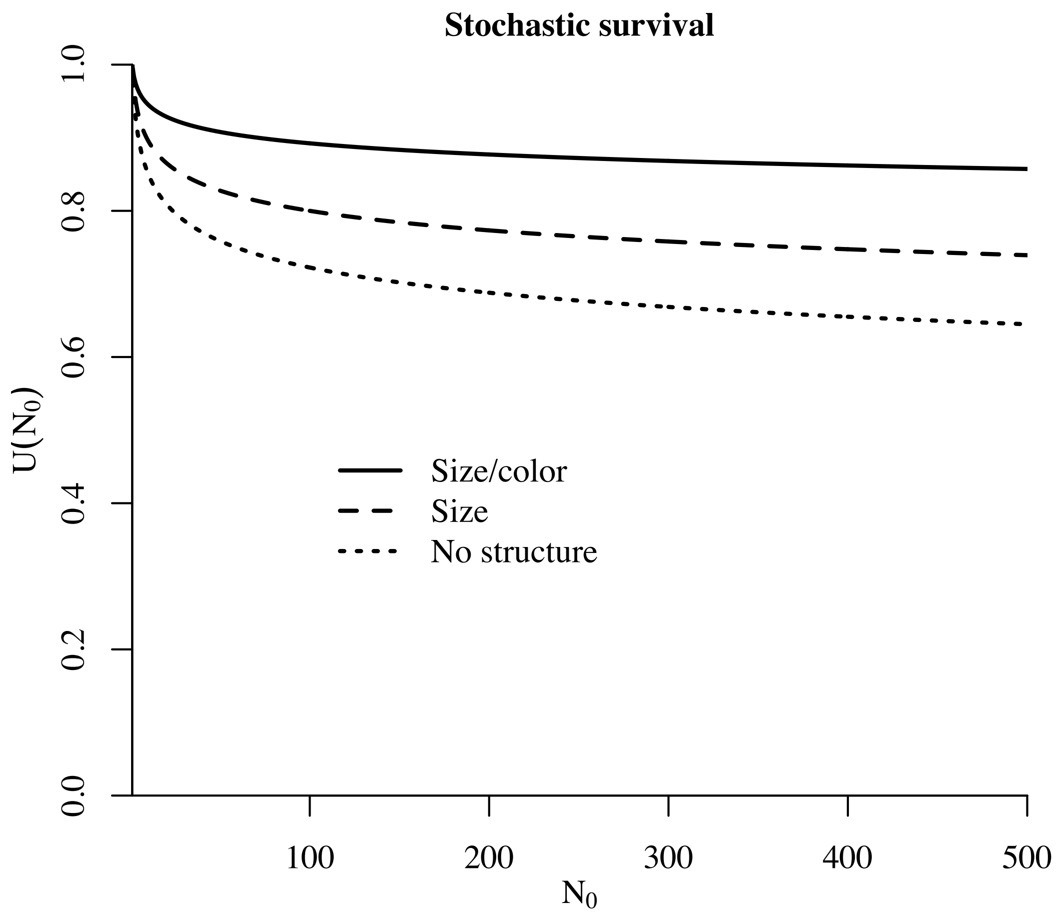

Figure 1: Example of calculated risk of ultimate extinction at $N = 1$ based on diffusion approximation, for the size/color structured population described in the main text (figure 2), including demographic stochasticity and environmental stochasticity in survival (see details in supplementary R code).

Appendix S3: Details on the pike model and study system

In this appendix we describe the derivation of the model for pike (*Esox lucius*) with individual heterogeneity in somatic growth and survival, and also provide some information about the study system and data. This model is an extension of the model of Vindenes *et al.* (2014) to include individual heterogeneity arising from early growth differences.

The model presented here is a one-sex model with two individual state variables, body length x and length at age 1 y, in addition to temperature T as environmental driver. Thus, in this particular case $x' = y'$ for offspring. We assume a pre-breeding census so that the fecundity function includes offspring survival. Each year we assume that an individual first reproduces, then, if it survives, grows to reach a new size next year.

S3-1. Study system and data

The study system is described in detail elsewhere (Le Cren, 2001; Winfield *et al.*, 2008; Vindenes *et al.*, 2014), here we provide a short summary. The pike data are collected at Windermere, which is situated in the English Lake District, UK (54°22′ N, 2°56′ W; altitude 39 m). Data have been collected since the 1940s, covering pike and other major fish species, as well as the abiotic and biotic environment (Le Cren, 2001; Winfield *et al.*, 2008). The annual mean surface temperature has shown a warming trend over the last decades (Winfield *et al.*, 2008; Vindenes *et al.*, 2014). Scientific monitoring of the pike population was initiated in 1944 with a removal program to enhance perch (*Perca fluviatilis*) numbers, and continues today with few changes in methods other than a much reduced sampling effort (Le Cren, 2001). Each fall/winter (in most years from

mid October to December), pike have been captured with 64 mm mesh gillnets in shallow areas of the lake. Captured pike were killed, measured for body length (in centimeters, measured as fork length), weighed (in kilograms), and sexed. Opercular bones were removed for age and length back-calculation, following a method validated for Windermere pike by Frost & Kipling (1959). Since 1963, data on female reproductive investment (gonad weight, egg number, and egg weight) have also been collected (Frost & Kipling, 1967). In this study we use body length estimates that are measured and back-calculated using catch data for females from 1946-1990 (7939 individuals).

The survival data set (Winfield *et al.*, 2013c) contains survival data from 1953-1990, for both males and females (3992 individuals), based on the capture mark recapture (CMR) study (for details on this study see Kipling & Le Cren, 1984; Haugen *et al.*, 2007). There is no age information on these individuals, so survival is assumed to depend on length only. Data from both males, females and individuals of unknown sex (in total 3992 individuals) are used, in order to increase the statistical strength of the estimation procedure. Analysis of this data set is complicated by the fact that for individuals who were never recaptured, the time of death and therefore also the length at death are unknown (Windermere is a closed system so individuals do not leave the system). Vindenes *et al.* (2014) used a Monte Carlo resampling method to correct for this bias, including information from the somatic growth model.

The four data sets used by Vindenes *et al.* (2014) have been made available online (see URL for each data set in the references). They are i) Fecundity data (Winfield *et al.*, 2013a), ii) Growth data (Winfield *et al.*, 2013b), iii) Survival data (Winfield *et al.*, 2013c), and iv) Lake temperature data (Winfield & Fletcher, 2013). The extended model presented here is based on the same data.

S3-2. Vital rate functions

Estimation of the vital rates from data was done with mixed effects models using the software R (R Development Core Team, 2013). Model selection between candidate models was based on AIC, details are provided by Vindenes *et al.* (2014). The main extensions in this model are inclusion of size at age 1 (heterogeneity) in the growth model and the survival model. We also evaluated potential effects of size at age 1 for fecundity (egg number), but these were so small that they were excluded from further analyses.

Below we describe each of the four main vital rate functions included in the model. Table S3.1 summarizes the fixed effects and variance components, while the variance covariance matrix for the residual year effects is shown in table S3.2. The random year effects all have expectation zero, and are assumed to be multivariate Gaussian distributed in the stochastic calculations (Vindenes *et al.*, 2014).

Fecundity

Fecundity is defined as the number of 1-year old female offspring produced by a female of length x at the temperature T, and is given by

$$b(x,T) = 0.5 p_m(x) m(x,T) s_0,$$

where $p_m(x)$ is the probability of maturity, $m(x,T)$ is the number of eggs produced, and s_0 is the survival probability from egg to 1-year old. The factor 0.5 enters because the model is female based, assuming a 1:1 sex ratio. The function $m(x,T)$ describing egg numbers was estimated by (Vindenes *et al.*, 2014) and is given by

$$\sqrt{m(x,T)} = \beta_{m0} + \beta_{mx}x + \beta_{mT}T + \beta_{mxT}Tx + \varepsilon_m,$$

where ε_m is the random year effect for egg number. Data on offspring survival (from egg to age 1) is not available for this system, thus s_0 is a free parameter in the model. The value ($s_0 = 0.00018$) was chosen so that the population growth rate predicted by the model roughly matches the observed growth rate, as estimated by Langangen *et al.* (2011). This value is consistent with values reported from other studies (Wright, 1990). Egg survival is thus modeled as

$$\operatorname{logit} s_0 = \beta_{s0} + \varepsilon_{s0},$$

where ε_{s0} is the random year effect for egg survival (chosen to be normally distributed with standard deviation 0.1). The probability of maturity $p_m(x,T)$ is also modeled on a logit scale,

$$\operatorname{logit} p(x) = \beta_{p0} + \beta_{px}x.$$

The values of β_{p0} and β_{px} are chosen based on information from a study by Frost & Kipling (1967), who found that females mature at a size between 31-50 cm, with a mean size of 41.5. They also noted that in pike maturity is largely determined by size rather than age. Figure S3.1 shows the underlying functions and the resulting total fecundity $b(x,T)$.

The fecundity function presented here is slightly modified compared to the model of Vindenes *et al.* (2014) as here we included a reaction norm for size at

maturity (without large changes in the overall fecundity function) and included two subsequent temperatures as egg formation happens in the year preceding the spawning year, while survival from egg to age 1 depends on temperature in the spawning year. This has no consequences for the deterministic model (assuming constant temperature), and only small consequences for the stochastic model.

Offspring length distribution

Vindenes *et al.* (2014) estimated the offspring length distribution $f(y'; T)$ from back-calculated data, using a lognormal distribution where the mean $\mu_{L1}(T)$ depends on temperature but the variance σ_y^2 is constant. The mean (on absolute scale) is given by (Vindenes *et al.*, 2014)

$$\mu_y(T) = \beta_y + \beta_y T + \varepsilon_{L1},$$

where ε_{L1} is the random year effect for the offspring length distribution. The variance is constant. Figure S3.1 shows the distribution $f(y'; T)$ at three different temperatures.

If y has a heritable component, so that relatively larger parents (large y) tend to produce relatively larger offspring, this can be included in the offspring size distribution by adding an effect of parental size x in the mean, while reducing the variance σ_{L1}^2 accordingly.

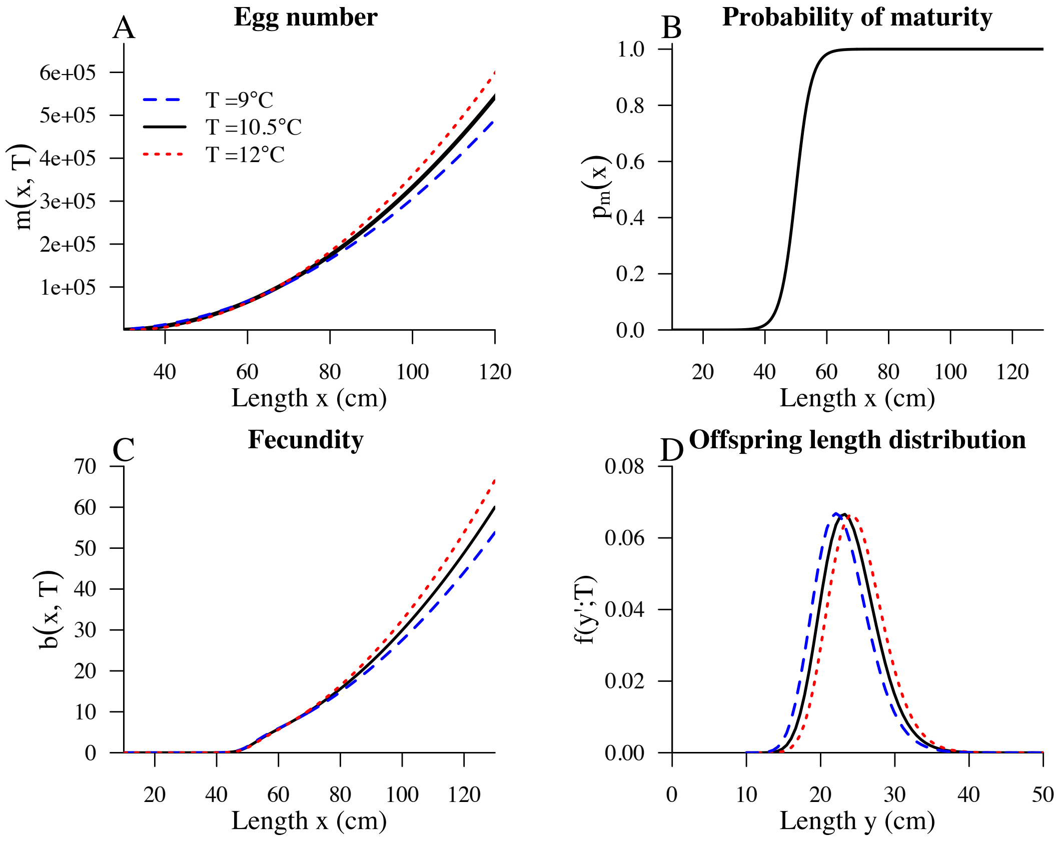

Figure S3.1: Fecundity functions and offspring size distribution for the pike model, shown for three different temperatures. Fecundity does not depend on the static trait y (length at age 1). A. Egg number $m(x,T)$. B. Probability of maturity $p_m(x)$. C. Total fecundity $b(x,T) = m(x,T)p_m(x)s_0$, where $s_0 = 0.00018$. D. Offspring length distribution, $f(y';T) = f(x';T)$.

Survival

The survival probability was estimated as a function of length and temperature by Vindenes *et al.* (2014). Here we use this function and also added an effect α of the static trait y, adjusting the intercept accordingly so that the average survival is the same as before. The survival model is then given by

$$\text{logit}\, s(x, y, T) = (\beta_{0s} - \alpha\bar{y}) + \beta_{xs}x + \beta_{Ts}T + \beta_{Txs}Tx + \alpha y,$$

where $\bar{y}$ is the average of y in the mean environment. Figure S3.2 shows the survival function for two values of α, a negative value corresponding to a trade-off between growth and survival, and a positive corresponding to an "individual quality" effect.

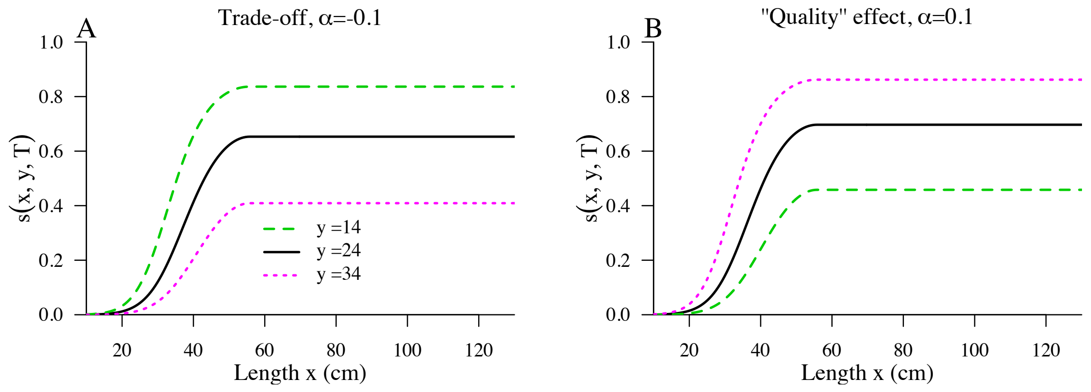

Figure S3.2: Survival as a function of length x shown for three values of y (length at age 1). The panels represent two scenarios for the effect of y on survival. A. A "trade-off" scenario, where rapid growth is associated with increased mortality (see also figure S3.3). B. A "quality effect" scenario, where individuals with rapid growth also have higher survival.

The parameter α measuring the effect of y on survival cannot be estimated from the CMR data. However, using the data from the winter fishery where age

as well as y is measured, we considered the effect of y on age at capture, to get some idea of whether the effect is present and the direction of the effect. This analysis shows that age at capture was negatively associated with y, which may indicate a trade-off between rapid early growth and later survival (figure S3.3). Increased early growth will also make individuals susceptible to the fishery at an earlier age, but the fishing pressure from this scientific fishery is so low that it seems unlikely that this could explains all of the effect on mortality. Thus, this analysis at least indicates that such a negative trade-off might be present, although it cannot be used to give a reliable measure of α for the survival model. In the results provided in the main text (also below) we therefore explore consequences of ignoring heterogeneity for different values of α ranging from strong negative values (trade-off effects) to positive ("individual quality" effects) values. This exercise also provides a good illustration of how the results depend on the degree of underlying heterogeneity (varying with α).

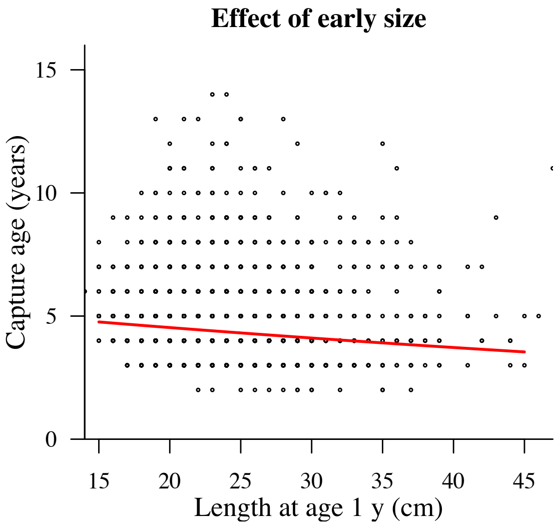

Figure S3.3: Effect of the static trait y (length at age 1) on age at capture. The estimated relationship (red line) is given by $\ln \text{Age} = 1.71 - 0.00984y$. This result is only included as an indication that there might be a trade-off between rapid growth and mortality, and is not used in the model.

Somatic growth

The somatic growth model measures the distribution of next year's length x' as a function of current length x, temperature T, and length at age 1 y. The mean and variance of this distribution was estimated by a mixed model including year as random effect and the other variables as fixed effects. Several candidate models were considered including second order effects of x, as well as interaction effects between x, y, and T. The variance of x' was modeled as an exponential function of current length x (with no effect of y and T). The final model (lowest AIC) for mean length is given by

$$\mu_G(x, y, T) = \beta_0 + \beta_x x + \beta_y y + \beta_T T + \beta_{x^2} x^2 + \beta_{xT} T + \beta_{xT} T + \beta_{xy} xy + \beta_{x^2 y} x^2 y + \varepsilon_G,$$

where ε_G is the random year effect, while the variance function is given by

$$\sigma_G^2(x, y, T) = \tau_g e^{\nu_g x},$$

where $\tau_g = 10.7$ and $\nu_g = -0.0061$. The resulting model for mean length next year includes an interaction between x and y as well as an interaction between x^2 and y (see figure S3.4). This second order interaction occurs because of stronger non-linearity in the growth trajectory of individuals of small y, while those of large y have more linear trajectories. The growth data thus suggest that initially small individuals increase their growth rates more at low sizes (an "attempt to catch up" with the larger ones), but then stop growing at a smaller final size, while the larger individuals show a more steady decline in the growth rate over their lifetime.

The final growth distribution $g(x'; x, y, T)$ is modeled as a lognormal distribution with mean $\mu_G(x, y, T)$ and variance $\sigma^2_G(x, y, T)$, truncated to zero for $x' < x$ to prevent individuals from shrinking in length (Vindenes *et al.*, 2014).

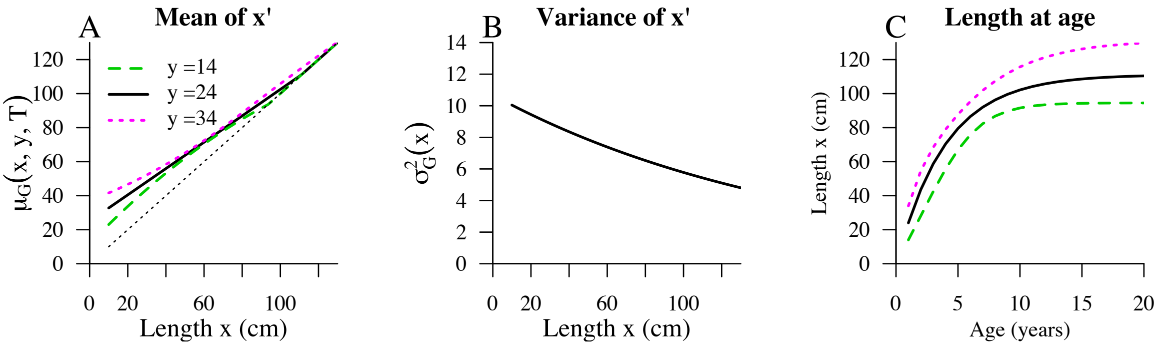

Figure S3.4: A. Mean size next year x' as a function of current size, for $T = 10.5°C$ and for three values of the static trait y (length at age 1). B. Variance of next year's size as a function of current size. C. Expected length at age (conditional on survival) based on the growth function shown in A.

Table S3.1: Fixed effects of the vital rate functions for the pike example. Where estimated, standard errors are provided in parentheses. Vital rates not estimated from data are denoted by an asterisk, the effect α is a parameter to be varied in the model.

	$\sqrt{m(x,T)}$	logit $S(x,y,T)$	$\mu_G(x,y,T)$	$\mu_{G1}(T)$	$\ln\sigma^2_G(x)$	σ^2_{G1}	logit $p^*_m(x)$	logit s^*_0
β_0	73.3(73.5)	−13.53(3.74)	−14.9(11.97)	16.9(22.0)	2.37	13.8	−20	−13.53
β_x	3.38(0.97)	0.510(0.038)	1.70(0.027)		−0.0061		0.4	
β_x^2		−0.004(0.000)	−0.007(0.000)					
β_y		α	1.21(0.026)					
β_T	−28.3(6.9)	0.193(0.175)	1.036(0.159)	0.669(0.273)				
β_{xy}			−0.0354(0.0011)					
β_{xT}	0.44(0.09)	−0.007(0.003)	−0.008(0.001)					
β_{x^2y}			0.00027(0.00001)					

Table S3.2: Variance covariance matrix for the random year effects (following Vindenes *et al.*, 2014). Egg survival to age 1 s_0^* is not estimated from data and covariance with other vital rates is set to zero.

	$\sqrt{m(x,T)}$	$\text{logit}\, S(x,y,T)$	$\mu_G(x,y,T)$	$\mu_{G1}(T)$	$\text{logit}\, s_0^*$
$\sqrt{m(x,T)}$	155.949	0.262	1.721	4.841	0
$\text{logit}\, S(x,y,T)$	0.262	0.010	−0.001	0.015	0
$\mu_G(x,y,T)$	1.721	−0.001	0.281	0.240	0
$\mu_{G1}(T)$	4.841	0.015	0.240	0.914	0
$\text{logit}\, s_0^*$	0	0	0	0	0.1

S3-3. Projection kernel and results

For a given temperature T, the projection kernel for this model is given by

$$K(x', y'; x, y, t) = s(x, y, T)g(x'; x, y, T)\delta(y' - y) + f(y'; T)\delta(x' - y')b(x, T).$$

In this particular example the distributions of the dynamic and static traits are exactly the same at age 1, therefore a delta function also enters the second term corresponding to the reproduction kernel. This ensures that if $x' \neq y'$ for offspring, the probability of offspring obtaining a length combination x', y' is zero.

To evaluate consequences of environmental stochasticity, we generate a sequence of projection kernels from the model. Here the stochasticity arises from temperature (a normal distribution; Vindenes *et al.*, 2014) and through residual variation and covariation between the year effects of the estimated vital rates (variance/covariance matrix provided in R code, further details provided by Vindenes *et al.*, 2014). These residuals were simulated using a multivariate Gaussian distribution. In the stochastic IPM the effects of temperature occurs at different time steps. Egg number depends on temperature the year before (as egg

formation occurs in the female the autumn before the spawning year) while all other temperature-dependent vital rates depend on temperature of the current year. This was not included in the original model of Vindenes *et al.* (2014) but has very small effects on the results.

Reduced model treating y as a parameter

If y is treated as a parameter rather than a state variable (which is causing some conflicting implications as it implicitly assumes perfect heritability of y but at the same time allowing lengths at age 1 x different from y) the resulting model is only length-structured (not to be confused with the model ignoring y used to evaluate consequences of ignoring heterogeneity). Figure S3.5 shows the population growth rate λ as a function of y in the reduced model, for three different values of α. This result suggests that when a growth/survival trade-off is present (negative α) the average fitness declines with y (unless the trade-off is very weak), otherwise larger values of y leads to a higher fitness. This model is only an approximation as the assumptions are not realistic, but can be a useful first step to investigate the dynamics.

Stable structure and reproductive values in full model

Figure S3.6 shows the stable trait distribution and reproductive value for three values of α (determining the survival effect of y). For negative values of α there is a growth/survival trade-off, and here individuals of small y have a higher reproductive value than those of large y, in particular for larger lengths x. For positive values of α, large y individuals with more rapid growth also have higher survival, and here they also have the highest reproductive value. For this pike population the scenario of a trade-off is more likely (figure S3.3). With no effect of y on survival ($\alpha = 0$) the differences in reproductive value across y are small

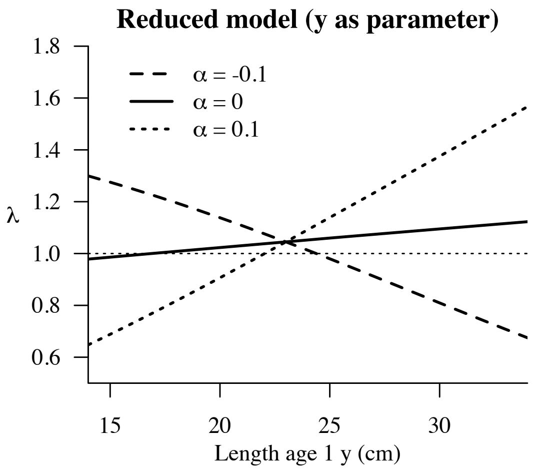

Figure S3.5: Effect of y on long-term growth rate λ in the reduced model treating y as a parameter rather than a state variable (assuming all individuals have the same y value), estimated for the mean temperature $T = 10.5$°C. The different lines represent different assumptions of the effect of y on survival, where $\alpha = -0.1$ corresponds to a growth/survival trade-off, $\alpha = 0$ represents the case of no effect (y only affects growth) and $\alpha = 0.1$ represents a case of a "quality effect" (individuals with rapid growth also have higher survival).

(pike with larger y have a slightly larger reproductive value at intermediate sizes). Note that age differences in reproductive value will still be large, as pike with large y will reach a given size x at a younger age than individuals of low y. Thus, there are still large individual differences, but because they only affect growth they are largely captured by the dynamic trait x.

Effects of ignoring heterogeneity in y

Table S3.3 shows the resulting demographic outputs of the full model and a model ignoring y, calculated for different values of α. These values were used in figure 3 of the main text, where the results are also discussed.

Table S3.3: Outputs from the pike model with individual heterogeneity in the static trait y (length at age 1), and a model ignoring this heterogeneity, under different values of the parameter α determining effect of y on survival. The percentage difference is also shown for each case ($100(M_2-M_1)/M_1$, where M_1 is the parameter from the heterogeneous model while M_2 is the corresponding parameter from the model ignoring y). The differences are smallest for α values near zero, where y only affects somatic growth.

**Value of α**	**Model output**	**Full model**	**Ignoring y**	**% difference**
$\alpha = -0.1$	λ	0.942	0.942	0
	R_0	0.680	0.690	1.403
	G_1	6.483	6.249	3.619
	G_2	6.913	6.607	-4.425
	σ_d^2	4.512	4.647	2.981
	σ_e^2	0.0053	0.0078	46.151
$\alpha = -0.05$	λ	0.989	0.989	0
	R_0	0.933	0.934	0.122
	G_1	6.037	5.930	-1.757
	G_2	6.092	5.981	-1.826
	σ_d^2	4.068	4.150	2.000
	σ_e^2	0.0054	0.0062	14.098
$\alpha = 0$	λ	1.074	1.074	0
	R_0	1.550	1.553	0.230
	G_1	6.113	6.141	0.463
	G_2	5.792	5.815	0.413
	σ_d^2	3.821	3.801	-0.534
	σ_e^2	0.0069	0.0068	-2.660
$\alpha = 0.05$	λ	1.184	1.184	0
	R_0	3.065	3.168	3.342
	G_1	6.639	6.834	2.935
	G_2	5.734	5.832	1.715
	σ_d^2	3.409	3.370	-1.156
	σ_e^2	0.0082	0.0074	-9.127
$\alpha = 0.1$	λ	1.294	1.294	0
	R_0	6.758	7.424	9.849
	G_1	7.422	7.787	4.916
	G_2	5.674	5.791	2.060
	σ_d^2	2.786	2.814	1.033
	σ_e^2	0.0113	0.0099	-12.020

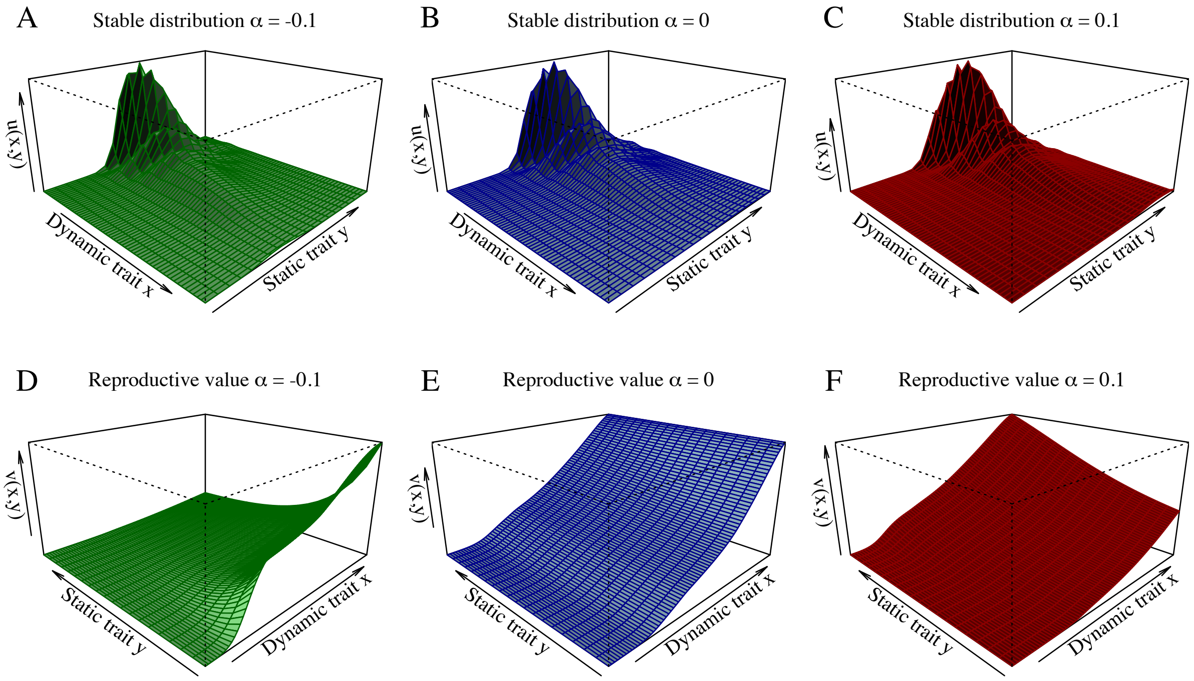

Figure S3.6: Stable trait distribution (A-C, smallest lengths x corresponding to age 1 not shown) and reproductive value (D-F) for three values of α, where $\alpha = -0.1$ (A,D) corresponds to a growth/survival trade-off scenario ($\lambda = 0.942$), $\alpha = 0$ (B,E) represents the case where y affects growth only ($\lambda = 1.074$) and $\alpha = 0.1$ (C,F) represents a "quality effect" (rapid growth is associated with higher survival, $\lambda = 1.294$). The spiky points at low lengths in the stable distribution occur because $x' = y'$ for offspring, while after the first year there is a spread in x-values for each y.

Appendix S4: Eco-evolutionary dynamics with discrete trait (color example)

Here we provide an example of eco-evolutionary dynamics for a model with a discrete static trait with an explicit mechanism for genetic inheritance. This model is based on the theoretical example of the size-structured population (dynamic trait size x) of red and green individuals, introduced in the main text.

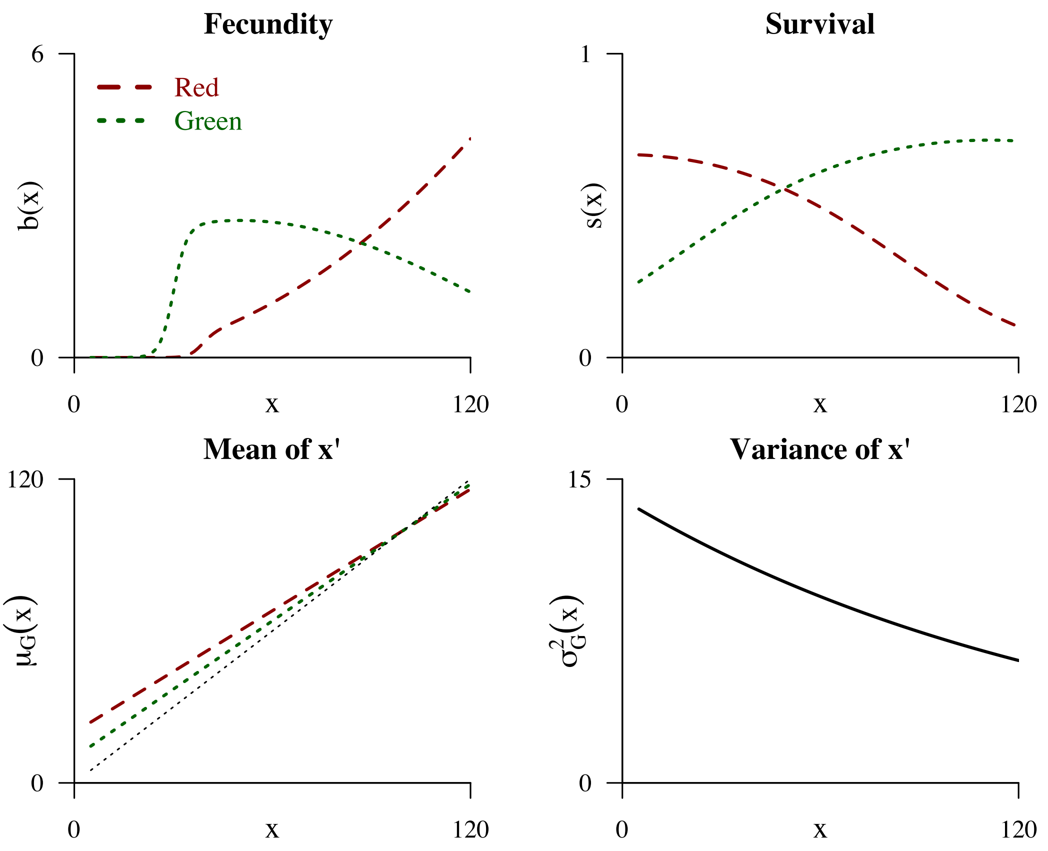

Figure S4.1: Fecundity, survival, and mean and variance of the growth function (of size x) used in the theoretical example of red and green individuals.

S4-1: Vital rates, effect of environment and harvesting

The survival probability, fecundity, and mean and variability of the somatic growth rate (transition in x) of the model are shown in figure S4.1. We now include an environmental variable θ that has opposite effect on survival of red and green individuals, as shown in figure S4.2. Thus, for a given range of θ green individuals will be favored towards one end while red individuals are favored towards the other, with an unstable equilibrium at an intermediate value. Using this model we will also consider a case of harvesting, where a constant proportion of the population above a certain body size (both colors) will be be harvested. This is also included in the survival function.

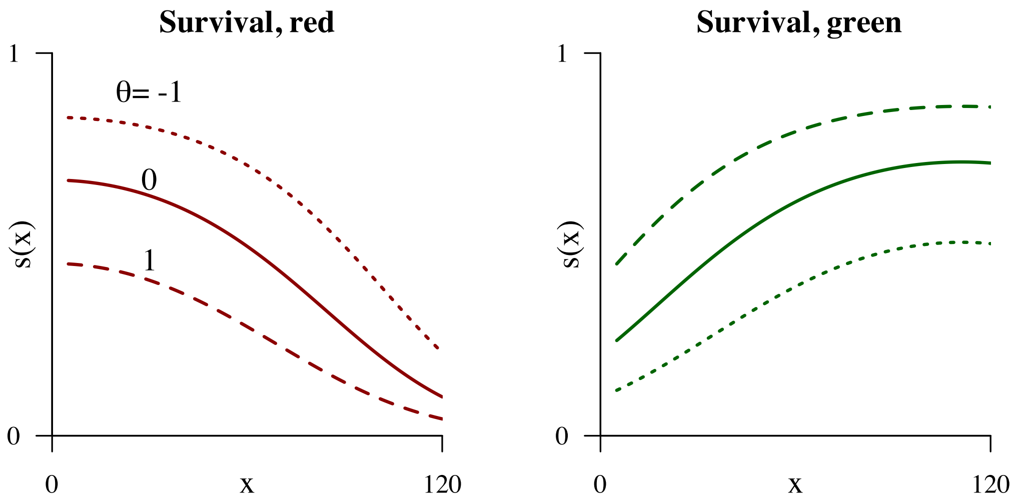

Figure S4.2: Effect of environmental variable θ (shown for three different values), on survival or red and green individuals.

S4-2: Genetic mechanism of inheritance of color

We will compare a model where color is genetically determined to the model used in example 1 of the main text, where color is randomly assigned at birth with

probability 0.5 (an arbitrary selected value). The genetic model assumes a Mendelian mechanism of inheritance with three genotypes determined by two alleles (A and a) on one locus. Genotypes AA and Aa both produce the red phenotype, while genotype aa produces the green phenotype.

The model is female based, and we assume that the genotype distribution is the same in males and females (i.e., males and females have the same life history). Females reproduce once per year and choose a mate randomly from the population. Let r be the frequency of red individuals in the population, while p is the frequency of allele A. Since p is not observed, we aim to express the offspring color distribution as a function of r, for each color of parent. Using the Hardy Weinberg frequencies of the genotypes (p^2, $2p(1-p)$ and $(1-p)^2$), the relationship between p and r is given by

$$
\begin{aligned}
r &= p^2 + 2p(1-p) \\
p &= \frac{2 \pm \sqrt{4-4r}}{2} = 1 - \sqrt{1-r},
\end{aligned}
$$

where the other solution is not applicable as $0 < p < 1$. Let O_R and O_G denote the events that an offspring is red and green, respectively, while M_R and M_G denote the events of a red and green parent. Further, let M_{AA} denote the event that a parent has genotype AA, and similarly for the other two genotypes. The conditional probabilities of offspring color given parental (maternal) color are then given by

$$\begin{aligned} P(O_R|M_R) &= P(O_R|M_{AA})P(M_{AA}|M_R) + P(O_R|M_{Aa})P(M_{Aa}|M_R) \\ &= \frac{p^2}{r} + \frac{2p(1-p)}{r}\left[p^2 + \frac{3}{4}2p(1-p) + \frac{1}{2}(1-p)^2\right] \\ &= \frac{1+p(1-p)}{2-p}, \end{aligned}$$

$$\begin{aligned} P(O_G|M_R) &= P(O_G|M_{AA})P(M_{AA}|M_R) + P(O_G|M_{Aa})P(M_{Aa}|M_R) \\ &= 0 + \frac{2p(1-p)}{q}\left[0 + \frac{1}{4}2p(1-p) + \frac{1}{2}(1-p)^2\right] \\ &= \frac{(1-p)^2}{2-p}, \end{aligned}$$

$$\begin{aligned} P(O_R|M_G) &= P(O_R|M_{aa})P(M_{aa}|M_G) \\ &= p^2 + \frac{1}{2}2p(1-p) + 0 \\ &= p, \end{aligned}$$

and

$$\begin{aligned} P(O_G|M_G) &= P(O_R|M_{aa})P(M_{aa}|M_G) \\ &= 0 + \frac{1}{2}2p(1-p) + (1-p)^2 \\ &= 1-p. \end{aligned}$$

Inserting the expression for p as a function of r found above into these four

probabilities yields the conditional probabilities of offspring color as a function of parental color, expressed by the proportion of red individuals r.

S4-3: Effects of changing environment and harvesting

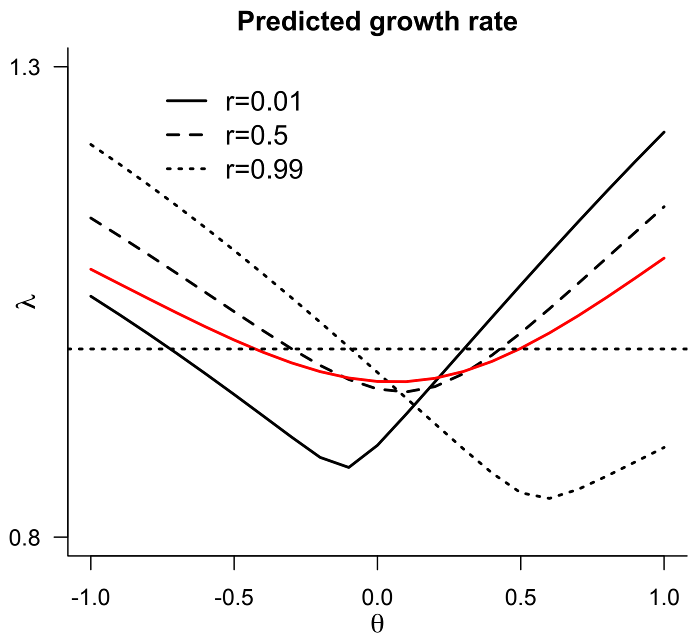

Figure S4.3: Predicted growth rate as a function of environment θ, for the non-genetic model with random assignment of color (red line) and the genetic model with Mendelian determination of color, for three values of r, the proportion of red individuals in the population.

Figure S4.3 shows the predicted long-term growth rate λ (average fitness) as a function of the environment θ, for the two models (genetic and non-genetic). For the intermediate values of the environment neither red or green individuals are well adapted and predicted growth rate is below 1. In the genetic model a population consisting of mostly red individuals has highest average fitness for low values of θ (for simplicity we call this a "red" environment), while for high values of θ a "green" population has highest fitness. The fitness of the "non-genetic"

population is also highest towards either end of the environmental range, but not as high as in the best adapted population with genetically determined color. The difference can be interpreted as the genetic load of having a higher variability of colors. In other words, local adaptation is generally higher in the case where color is genetically determined, but this comes at the cost of loosing color variation.

If the environment is now changing, for instance from a value that favors red individuals to one favoring green, the response time of the population will depend on the initial distribution of red and green individuals at the time of the change, but also on the generation time relative to the rate of environmental change. If generation time is long compared to the rate of change, the population with genetically determined color will be slower to respond than the population where color is randomly assigned.

Figure S4.4 shows an example where the environment suddenly changes from a value favoring red ($\theta = -0.4$) to one favoring green ($\theta = 0.6$) individuals, and the projected population growth in the models with random (non-genetic) and genetic determination of color. Both projections start from the same initial population of $N = 100$ at the predicted stable trait distribution for $r = 0.5$ in the genetic model. As the environment changes after a few time steps, the "genetic" population which had already become largely adapted to the red environment experiences a much larger decline than the "non-genetic" population, but then starts to increase and the growth rate of the population increases continually with the proportion of green individuals. The "non-genetic" population experiences only a small decline before increasing at a steady rate. Had the initial period of adaptation been a bit longer, the "genetic" population would not have had enough green individuals present (genetic variability) to respond to the change and would instead have gone extinct.

Many studies of eco-evolutionary dynamics focus on consequences of

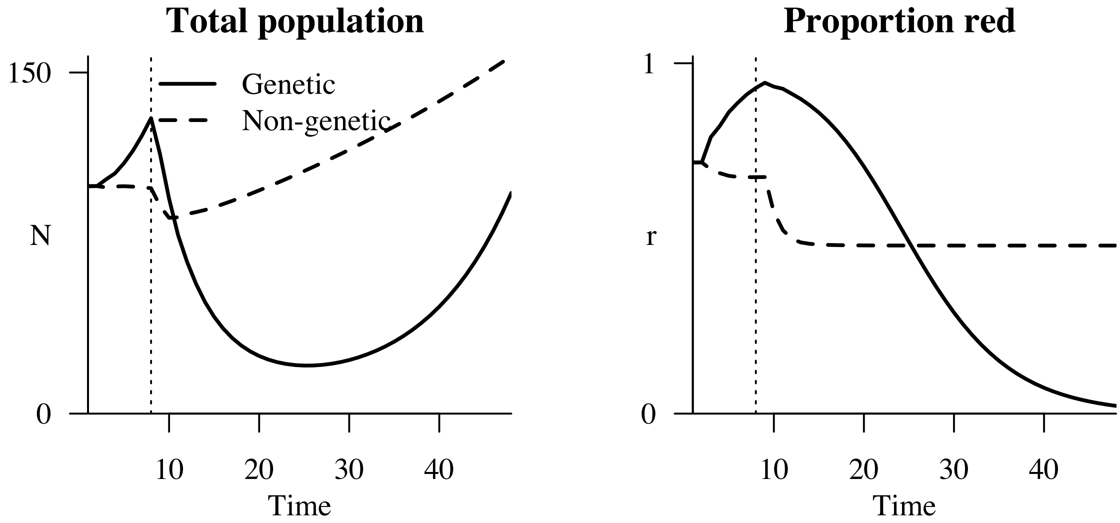

Figure S4.4: Projected changes in population size and color distribution through a sudden environmental shift, for a population where color is genetically determined ("Genetic") and a population where color is randomly assigned ("Non-genetic"). The populations both start from a density of 100 individuals at the stable distribution (estimated for the genetic model with $r = 0.5$). The environment has a value of $\theta = -0.4$ during the first time steps ("red" environment), then suddenly changes to a value of $\theta = 0.6$ ("green" environment) at the time indicated by the vertical dashed line.

harvesting, and in particular harvesting based on demographic traits such as size (e.g., Traill *et al.*, 2014). Therefore, we also considered what would happen in this example if we added proportional harvesting above some threshold size. Figure S4.5 shows the result for the same projections as in figure S4.4 (including the environmental change), but harvesting a proportion 0.8 of all individuals (both colors) above size $x = 60$. In this example, the growth rate of the "non-genetic" population becomes negative, leading the population process towards extinction, whereas the population with genetic heritability of color still manages to grow after a while, increasing the growth rate as the population is adapting to its new environment.

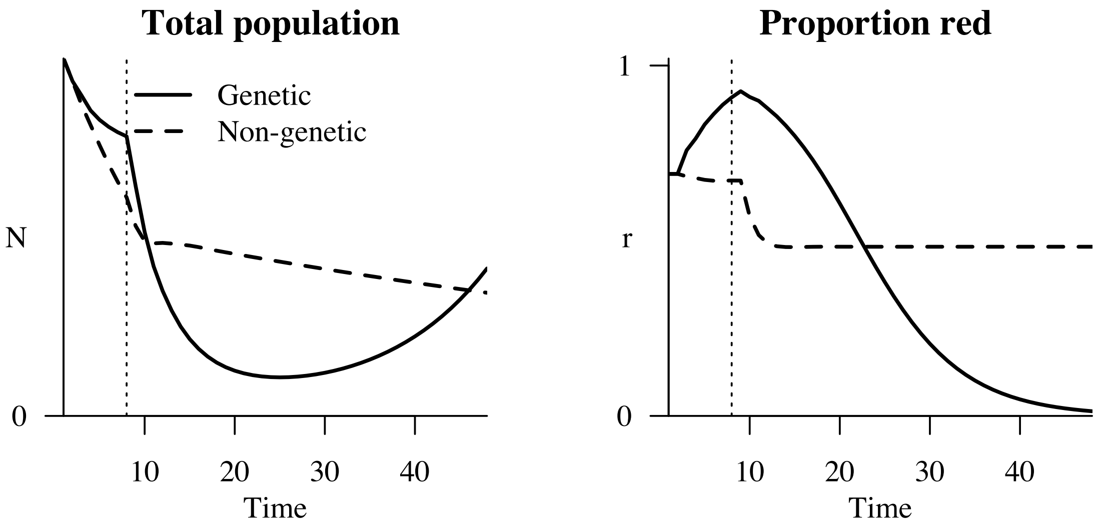

Figure S4.5: The same projected processes as in figure S4.4, with an added size-based harvesting pressure. Above a size of $x = 60$, a proportion of 0.8 of all individuals (both colors) are harvested.

References

Bartlett, M.S. (1960). *Stochastic population models.* Methuen & Co, London.

Bienvenu, F., Demetrius, L. & Legendre, S. (2013). A general formula for the generation time. *arXiv*, 1307.6692v3.

Caswell, H. (2001). *Matrix population models.* 2nd edn. Sinauer Associates, Sunderland, Massachusetts.

Cox, D. & Miller, H. (1965). *Theory of stochastic processes.* Methuen & Co, London.

Danchin, É. (2013). Avatars of information: towards an inclusive evolutionary synthesis. *Trends Ecol. Evol.*, 28, 351–358.

Easterling, M.R., Ellner, S.P. & Dixon, P.M. (2000). Size-specific sensitivity: applying a new structured population model. *Ecology*, 81, 694–708.

Ellner, S.P. & Rees, M. (2006). Integral projection models for species with complex demography. *Am. Nat.*, 167, 410–428.

Ellner, S.P. & Rees, M. (2007). Stochastic stable population growth in integral projection models: theory and application. *J. Math. Biol.*, 54, 227–256.

Engen, S., Bakke, Ø. & Islam, A. (1998). Demographic and environmental stochasticity -concepts and definitions. *Biometrics*, 54, 840–846.

Engen, S., Lande, R., Sæther, B.E. & Festa-Bianchet, M. (2007). Using reproductive value to estimate key parameters in density-independent age-structured populations. *J. Theor. Biol.*, 244, 308–317.

Engen, S., Lande, R., Sæther, B.E. & Weimerskirch, H. (2005). Extinction in relation to demographic and environmental stochasticity in age-structured models. *Math. Biosci.*, 195, 210–227.

Engen, S., Lande, R., Sæther, B.E. & Dobson, F.S. (2009). Reproductive value and the stochastic demography of age-structured populations. *Am. Nat.*, 174, 795–804.

Frost, W.E. & Kipling, C. (1959). The determination of the age and growth of pike (*Esox lucius* l.) from scales and opercular bones. *ICES J. Mar. Sci*, 24, 314–341.

Frost, W.E. & Kipling, C. (1967). A study of reproduction, early life, weight-length relationship and growth of pike, *Esox lucius* l., in Windermere. *J. Anim. Ecol.*, 36, 651–693.

Haugen, T.O., Winfield, I.J., Vøllestad, A., Fletcher, J.M., James, J.B. & Stenseth, N.C. (2007). Density dependence and density independence in the demography and dispersal of pike over four decades. *Ecol. Monogr.*, 77, 483–502.

Karlin, S. & Taylor, H.M. (1981). *A second course in stochastic processes*, Academic Press, New York.

Kendall, B.E., Fox, G.A., Fujiwara, M. & Nogeire, T. (2011). Demographic heterogeneity, cohort selection, and population growth. *Ecology*, 92, 1985–1993.

Kipling, C. & Le Cren, E.D. (1984). Mark-recapture experiments on fish in Windermere, 1943-1982. *J. Fish. Biol.*, 24, 395–414.

Lande, R., Engen, S. & Sæther, B.E. (2003). *Stochastic population dynamics in ecology and conservation.* Oxford University Press, Oxford.

Lande, R. & Orzack, S.H. (1988). Extinction dynamics of age-structured populations in a fluctuating environment. *Proc. Natl. Acad. Sci. U.S.A.*, 85, 7418–7421.

Langangen, Ø., Edeline, E., Ohlberger, J., Winfield, I.J., Fletcher, J.M., James, J.B., Stenseth, N.C. & Vøllestad, A. (2011). Six decades of pike and perch population dynamics in Windermere. *Fish. Res.*, 109, 131–139.

Le Cren, D. (2001). The Windermere perch and pike project: An historical review. *Freshw. Forum*, 15, 3–34.

Leslie, P.H. (1945). On the use of matrices in certain population mathematics. *Biometrika*, 33, 183–212.

Lewontin, R.C. & Cohen, D. (1969). On population growth in a randomly varying environment. *Proc. Natl. Acad. Sci. U.S.A.*, 62, 1056–1060.

May, R.M. (1973a). *Stability and complexity in model ecosystems.* Princeton University Press, Princeton, New Jersey.

May, R.M. (1973b). Stability in randomly fluctuating versus deterministic environments. *Am. Nat.*, 107, 621–650.

Merow, C., Dahlgren, J.P., Metcalf, C.J.E., Childs, D.Z., Evans, M.E.K. & Jongejans, E. *et al.* (2014). Advancing population ecology with integral projection models: a practical guide. *Methods Ecol. Evol.*, 5, 99–110.

R Development Core Team (2013). *R: A Language and Environment for Statistical Computing.* R Foundation for Statistical Computing, Vienna, Austria.

Rees, M., Childs, D.Z. & Ellner, S.P. (2014). Building integral projection models: a user's guide. *J. Anim. Ecol.*, 83, 528–545.

Rees, M. & Ellner, S.P. (2009). Integral projection models for populations in temporally varying environments. *Ecol. Monogr.*, 79, 575–594.

Traill, L., Schindler, S. & Coulson, T. (2014). Demography, not inheritance, drives phenotypic change in hunted bighorn sheep. *Proc. Natl. Acad. Sci. U.S.A.*, 111, 13223–13228.

Tuljapurkar, S. (1982). Population dynamics in variable environments. ii. Correlated environments, sensitivity analysis and dynamics. *Theor. Popul. Biol.*, 21, 114–140.

Tuljapurkar, S. (1990). *Population dynamics in variable environments.* Springer-Verlag, Berlin.

Turelli, M. (1977). Random environments and stochastic calculus. *Theor. Popul. Biol.*, 12, 140–178.

Vindenes, Y., Edeline, E., Ohlberger, J., Langangen, Ø., Winfield, I.J. & Stenseth, N.C. *et al.* (2014). Effects of climate change on trait-based dynamics of a top predator in freshwater ecosystems. *Am. Nat.*, 183, 243–256.

Vindenes, Y., Engen, S. & Sæther, B.E. (2008). Individual heterogeneity in vital parameters and demographic stochasticity. *Am. Nat.*, 171, 455–467.

Vindenes, Y., Engen, S. & Sæther, B.E. (2011). Integral projection models for finite populations in a stochastic environment. *Ecology*, 92, 1146–1156.

Vindenes, Y., Sæther, B.E. & Engen, S. (2012). Effects of demographic structure on key properties of stochastic density-independent population dynamics. *Theor. Popul. Biol.*, 82, 253–263.

Winfield, I.J. & Fletcher, J.M. (2013). Windermere lake temperature data 1944-2002. NERC Environmental Information Data Centre, http://dx.doi.org/10.5285/9520664c -eb4d-4700-b064-5d215d23e595.

Winfield, I.J., Fletcher, J.M. & James, J.B. (2013a). Pike fecundity data 1963-2002. NERC Environmental Information Data Centre, http://dx.doi.org/10.5285/b8886915-14cb-44df-86fa-7ab718acf49a.

Winfield, I.J., Fletcher, J.M. & James, J.B. (2013b). Pike growth data 1944-1995. NERC Environmental Information Data Centre, http://dx.doi.org/10.5285/637d60d6-1571-49af-93f7-24c1279d884d.

Winfield, I.J., Fletcher, J.M. & James, J.B. (2013c). Pike survival data 1953-1990. NERC Environmental Information Data Centre, http://dx.doi.org/10.5285/813e07dd-2135-49bc-93c6-83999e442b36.

Winfield, I.J., James, J.B. & Fletcher, J.M. (2008). Northern pike (*Esox lucius*) in a warming lake: changes in population size and individual condition in relation to prey abundance. *Hydrobiol.*, 601, 29–40.

Wright, R.M. (1990). The population biology of pike, *Esox lucius* L., in two gravel pit lakes, with special reference to early life history. *J. Fish. Biol.*, 36, 215–229.
